# Endosome mediated nucleocytoplasmic trafficking and endomembrane allocation is crucial to polyglutamine toxicity

**DOI:** 10.1007/s10565-024-09891-4

**Published:** 2024-06-20

**Authors:** Yuyu Nan, Wenfeng Chen, Fei Chen, Lili Wei, Aiyuan Zeng, Xiaohui Lin, Wenbin Zhou, Yufeng Yang, Qinghua Li

**Affiliations:** 1 Department of Neurology, Xiangya Hospital, Central South University, Changsha, 410000 China; 2 Department of Critical Care Units, The First Affiliated Hospital, Zhejiang University School of Medicine, Hangzhou, 311121 China; 3 Institute of Life Sciences, Fuzhou University, Fuzhou, Fujian Province 350108 China; 4 Guangxi Clinical Research Center for Neurological Diseases, Guilin, Guangxi 541001 China; 5 Department of Neurology, The Affiliated Hospital of Guilin Medical University, Guilin, Guangxi 541001 China; 6 Guangxi Key Laboratory of Brain and Cognitive Neuroscience, Guilin, Guangxi 541004 China

**Keywords:** Autophagy, Endosome, Inclusion body, Nucleocytoplasmic trafficking, Non-canonical role of ATG, Spinocerebellar ataxia

## Abstract

Aggregation of aberrant proteins is a common pathological hallmark in neurodegeneration such as polyglutamine (polyQ) and other repeat-expansion diseases. Here through overexpression of ataxin3 C-terminal polyQ expansion in *Drosophila* gut enterocytes, we generated an intestinal obstruction model of spinocerebellar ataxia type3 (SCA3) and reported a new role of nuclear-associated endosomes (NAEs)–the delivery of polyQ to the nucleoplasm. In this model, accompanied by the prominently increased RAB5-positive NAEs are abundant nucleoplasmic reticulum enriched with polyQ, abnormal nuclear envelope invagination, significantly reduced endoplasmic reticulum, indicating dysfunctional nucleocytoplasmic trafficking and impaired endomembrane organization. Consistently, *Rab5* but not *Rab7* RNAi further decreased polyQ-related NAEs, inhibited endomembrane disorganization, and alleviated disease model. Interestingly, autophagic proteins were enriched in polyQ-related NAEs and played non-canonical autophagic roles as genetic manipulation of autophagic molecules exhibited differential impacts on NAEs and SCA3 toxicity. Namely, the down-regulation of *Atg1* or *Atg12* mitigated while *Atg5* RNAi aggravated the disease phenotypes both in *Drosophila* intestines and compound eyes. Our findings, therefore, provide new mechanistic insights and underscore the fundamental roles of endosome-centered nucleocytoplasmic trafficking and homeostatic endomembrane allocation in the pathogenesis of polyQ diseases.

**Graphical Abstract:**

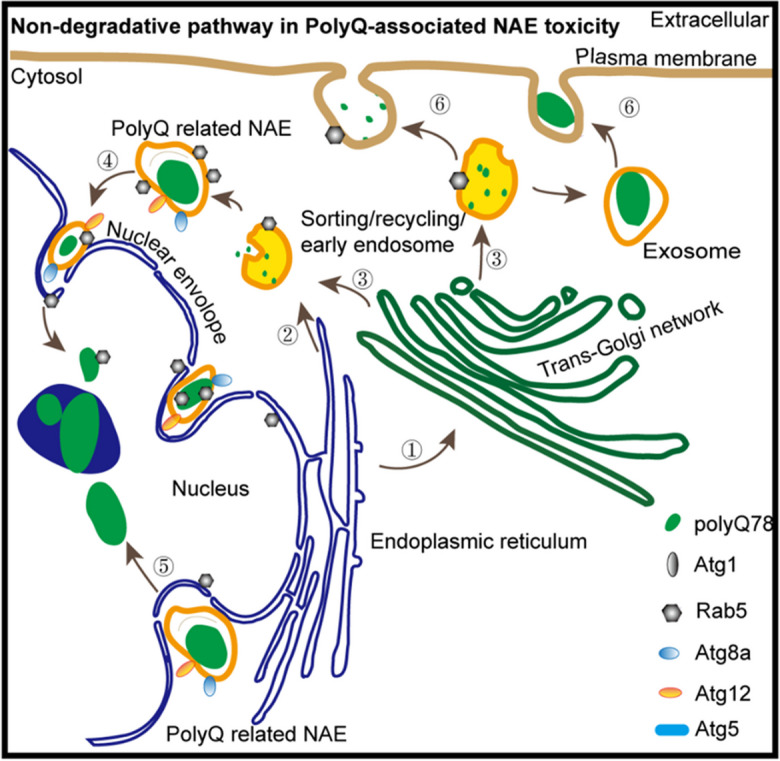

**Supplementary Information:**

The online version contains supplementary material available at 10.1007/s10565-024-09891-4.

## Introduction

Polyglutamine (polyQ) disorders encompass a spectrum of neurodegenerative conditions marked by the aberrant expansion of CAG trinucleotide repeats. This expansion leads to the synthesis of mutant proteins characterized by prolonged polyglutamine tracts. A unifying feature among PolyQ diseases and other repeat-expansion diseases is the formation of autophagy machinery positive intracellular inclusions in the cytoplasm and/or nucleus (Havel et al. 2009; Tian et al. 2019; Zhang et al. 2019). Notable examples within this disease group include Huntington's disease (HD), various spinocerebellar ataxias (SCAs), dentatorubral-pallidoluysian atrophy (DRPLA), and spinal and bulbar muscular atrophy (SBMA)(Lieberman et al. 2019). A profound comprehension of the pathogenic mechanisms underlying PolyQ disorders is indispensable for delineating their etiology and devising potential therapeutic interventions. Many reports suggest that both normal polyQ or aggregating expansions in the nucleus affecting disease onset or progression (Benn et al. 2005; Bichelmeier et al. 2007; Chan et al. 2011; Hernandez-Carralero et al. 2023), causing cytoplasmic organelle dysfunction (Benn et al. 2005), inhibiting nucleocytoplasmic trafficking (Gasset-Rosa et al. 2017). Recent studies on monomers, oligomers, cytoplasmic, intranuclear aggregates and even perinuclear aggregates(Liu et al. 2015) have revealed complex heterogeneity in the polyQ pathogenicity (Elena-Real et al. 2023; Kitamura and Kubota 2010; Trushina et al. 2003).

In many PolyQ diseases, polyQ preferentially accumulates in the nucleus to form nuclear inclusions (Havel et al. 2009). Trans-localization of proteins to the nucleus is achieved by passive nuclear pore (NP) transport of proteins with lower molecular weight, the nuclear pore complex (NPC)-dependent active transport pathway, and endosomal trafficking (Bocock et al. 2010; Li et al. 2014; Wing et al. 2022). Whether partial polyQ species are partially imported through the non-NPC pathway is still an open question (Desmond et al. 2012; Xia et al. 2003). Moreover, as the disease progresses in different models, NPC components decrease significantly and are mis-localized in the intranuclear inclusions, resulting in compromised NPC-dependent transport (Fahrenkrog and Harel 2018; Grima et al. 2017; Hutten and Dormann 2019; Sowa et al. 2018). Even under such conditions of severe NPC dysfunction, polyQ is still able to preferentially localize in the nucleus, which raises the possibility of the existence of a non-NPC-dependent route in the polyQ nucleocytoplasmic trafficking. Endosomes were known to serve as important trafficking bridges between different structures including the nucleus, plasma, endoplasmic reticulum (ER), lysosomes etc., which facilitate cargo trafficking and signal communication (Chaumet et al. 2015).The nuclear envelope-associated endosomal pathway, which can transport cell surface receptors such as EGFR, cellular E3 ubiquitin ligase and tumor-associated extracellular vesicle-derived biomaterials to the nucleus, could be an alternative trafficking route (Bocock et al. 2010; Chaumet et al. 2015; Raices and D'Angelo 2012; Rappa et al. 2017). Aberrant endomembrane organization has been implicated in polyQ-mediated toxicity, such as excessive endosomal structure accumulation (Sittler et al. 2018), ER, nuclear and plasma membrane disorganization (Gasset-Rosa et al. 2017; Skinner et al. 2001; Ueda et al. 2016). However, whether and how endosomes are related to polyQ preferential nucleocytoplasmic distribution and toxicity remains elusive.

In some nuclear inclusion diseases, inclusions are widely distributed in the skin and viscera, which may be accompanied by an intestinal obstruction (Clarke et al. 2007; Ferrer-Inaebnit et al. 2020; Rujano et al. 2006; Tian et al. 2019). Furthermore, it has been only recently reported that the presence of polyQ aggregates in committed crypt cells in the small intestine of SCA patients or animals (Clarke et al. 2007; Ferrer-Inaebnit et al. 2020; Rujano et al. 2006; Tian et al. 2019). As one of the most prone tissues of nuclear inclusions, the intestinal tract may be as suitable for studying the underlying pathogenesis as the nervous system. Towards this end, the genetically tractable *Drosophila* intestine — which is simpler than its vertebrate counterparts but has a similar anatomical and physiological function—has emerged as an advantageous alternative. In the present study, we overexpressed truncated spinocerebellar ataxia type 3 (SCA3) in *Drosophila* enterocyte cells (ECs) and developed an intestinal obstruction model. Using this model, we identified nuclear-associated endosomes (NAEs) as potential vehicles for pathological polyQ proteins and revealed disease modifying genes on the pathogenic pathway, thereby proposed an endosome-centered polyQ pathogenic mechanism, implicating a new avenue for the treatment of poly-expansion and other neurodegenerative diseases.

## Results

### Nuclear-associated aggregates formed in a SCA3 Drosophila intestinal obstruction model

**Fig. 1:**
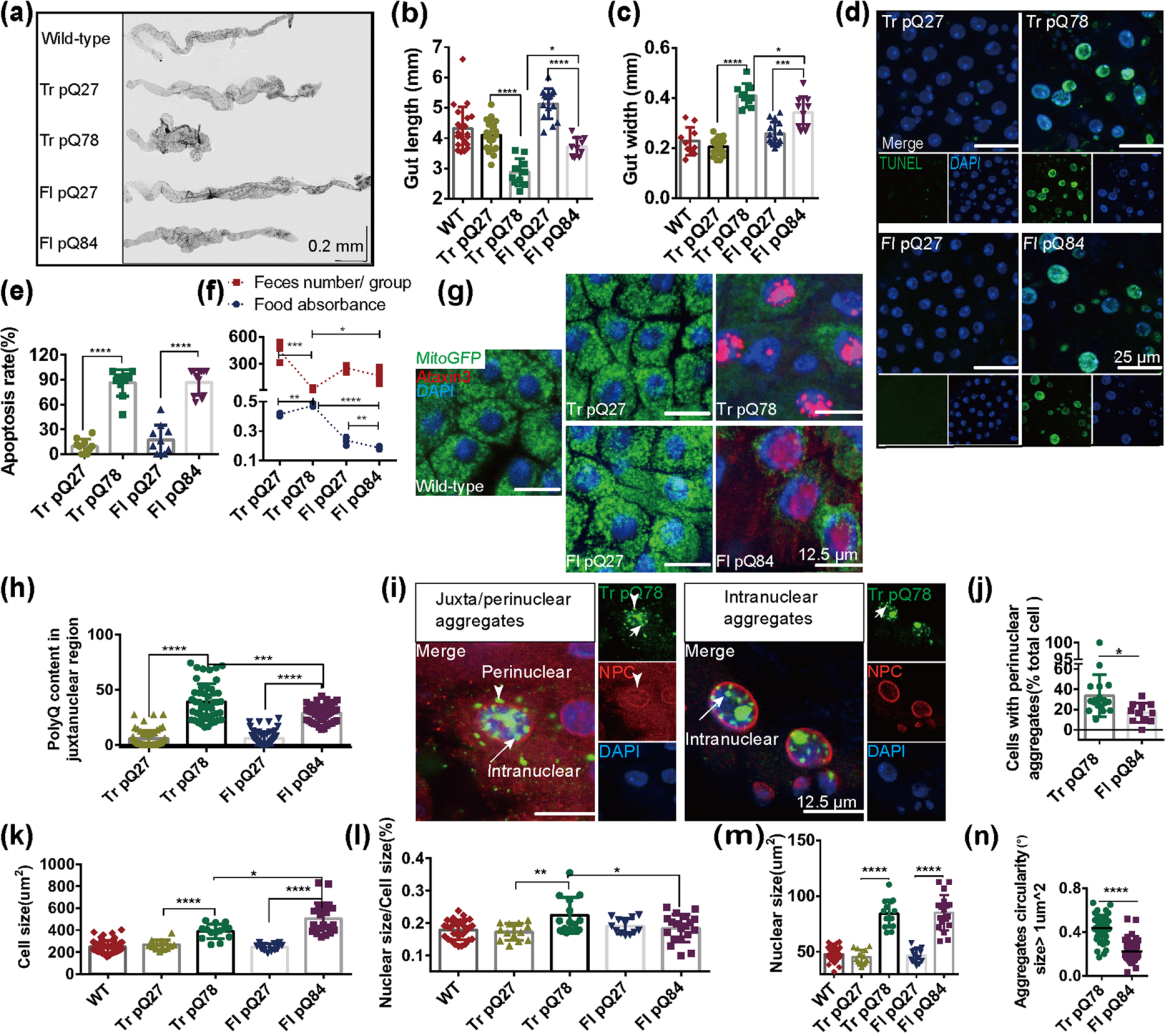
Nuclear-associated aggregates formed in a SCA3 *Drosophila* intestinal obstruction model **a** The gut morphology of the wild-type genetic background control *Drosophila* and those with enterocyte (EC)-specific overexpression of full length (fl) or truncated (tr) ataxin3pQ27 or pQ78 driven by NP1-Gal4. **b, c** The lengths (**b**) and width (**c**) of guts in panel (**A**), *n* ≥ 8. **d, e** Significantly higher TUNEL apoptotic rate in trpQ78 or flpQ84-expressing flies compared with that of pQ27. Nuclei were stained with DAPI. **f** Feeding and defecation capacity of the diseased (trpQ78 and flpQ84) and control flies (pQ27). TrpQ78 but not flpQ84 inhibited intestinal excretion and induced food retention. *n* = 3 groups, 20 flies per group. **g** Confocal microscopy of ataxin3 staining in the wild-type genetic background control intestinal ECs and ECs expressing trpQ27-HA, trpQ78-HA, flpQ27-myc, or ataxin3flpQ84-myc. Ataxin3 was immunostained with anti-HA or anti-myc antibody, respectively. Nuclei were visualized by DAPI staining. Multiple discrete juxtanuclear aggregates with clear boundaries were detected in trpQ78-HA-expressing ECs. By contrast, ataxin3 staining was in a fuzzy and continuous pattern in the nuclei with prominent intranuclear aggregates in flpQ84 ECs. **h** Comparison of the levels of ataxin3trpQ27, trpQ78, flpQ27 or flpQ84 in the perinuclear/juxtanuclear region (the maximum radial distance between the perinuclear region and the nuclear membrane was set to be one minimal nuclear semidiameter). **i** Confocal illustration of juxtanuclear/perinuclear and intranuclear polyQ aggregates in trpQ78 expressing ECs. Nuclear membrane was stained with an anti-NPC antibody. NPC = nuclear pore complex. **j** Percentages of cells with perinuclear aggregates in trpQ78 or flpQ84-exressing groups. **k–m** Quantitative analysis of average cell size (**k**, marked by mitoGFP), ratio of nuclear size to cell size (**l**) and nuclear size (**m**, marked by DAPI), and in different model flies. **N** Aggregates in trpQ78 expressing ECs were rounder with higher circularity than those in flpQ84 group. For immunostaining test, at least 4 intestines were randomly chosen from each group, and 3 fields of view were randomly selected for each intestine. All values represent mean ± SD. *p < 0.05, **p < 0.01, ***p < 0.001, *p* < 0.0001 by One-way ANOVA (**b–e, h, k–m**) and Student’s t test (**f, j, n**)

Visceral organ inclusions and intestinal obstruction are common features of neuronal intranuclear inclusion diseases (Clarke et al. 2007; Schuffler et al. 1978; Sone et al. 2016). Interestingly, ectopical expression of either ataxin3trpQ78 (HA-tagged truncated C-terminal fragment of the human ataxin3 with a 78 repeat polyglutamine tract) or ataxin3flpQ84 (N-terminally myc-tagged full length human ataxin3 with a polyQ repeat of 84 amino acids), but not ataxin3flpQ27 (flpQ27) or ataxin3trpQ27 (trpQ27) specifically in ECs of *Drosophila* guts driven by NP1-Gal4, induced significant gut shortening and broadening (Fig. 1a–c). Previous studies suggested that pathological polyQ aggregates could induce apoptosis (Ferreira et al. 2023). In our study, the groups with trpQ78 or flpQ84 showed higher apoptotic rates (Fig. 1d, e). TrpQ78 but not flpQ84 inhibited intestinal excretion and induced food retention and thus induced severe intestinal obstruction (Fig. 1 a–c, f).

Immunofluorescence staining showed that nuclear-associated aggregates (NAAs, aggregates in the intranuclear and/ or perinuclear regions) of flpQ84 were enriched in the nucleus in a relatively continuous pattern, while trpQ78 proteins formed much more prominent juxtanuclear or perinuclear polyQ aggregates in addition to their intranuclear presence (Fig. 1g–j). Cellular and nuclear sizes were also measured (Fig. 1k–m): flpQ84-expression increased nuclear and cell size proportionally, while trpQ78 expression significantly increased nuclear size such that the nucleus to cell size ratio was elevated (Fig. 1l). Measurement of circularity, a commonly used indicator of the shape of a structure, indicated that in the trpQ78-expressing group, NAAs had more regular shape than those in the flpQ84-expressing group (Fig. 1n). These findings were consistent with the previous reports that truncated polyQ resulted in higher degree of pathogenicity (Haacke et al. 2006).

Given that the much more severe intestinal obstruction phenotype and the prominent perinuclear aggregates observed in trpQ78-expressing flies, the SCA3-mediated intestinal obstruction *Drosophila* model was mainly based on trpQ78 in follow up studies, with corresponding genetic background or trpQ27 being used as the control.

### Truncated polyQ enriched in nuclear-associated endosomes

**Fig. 2:**
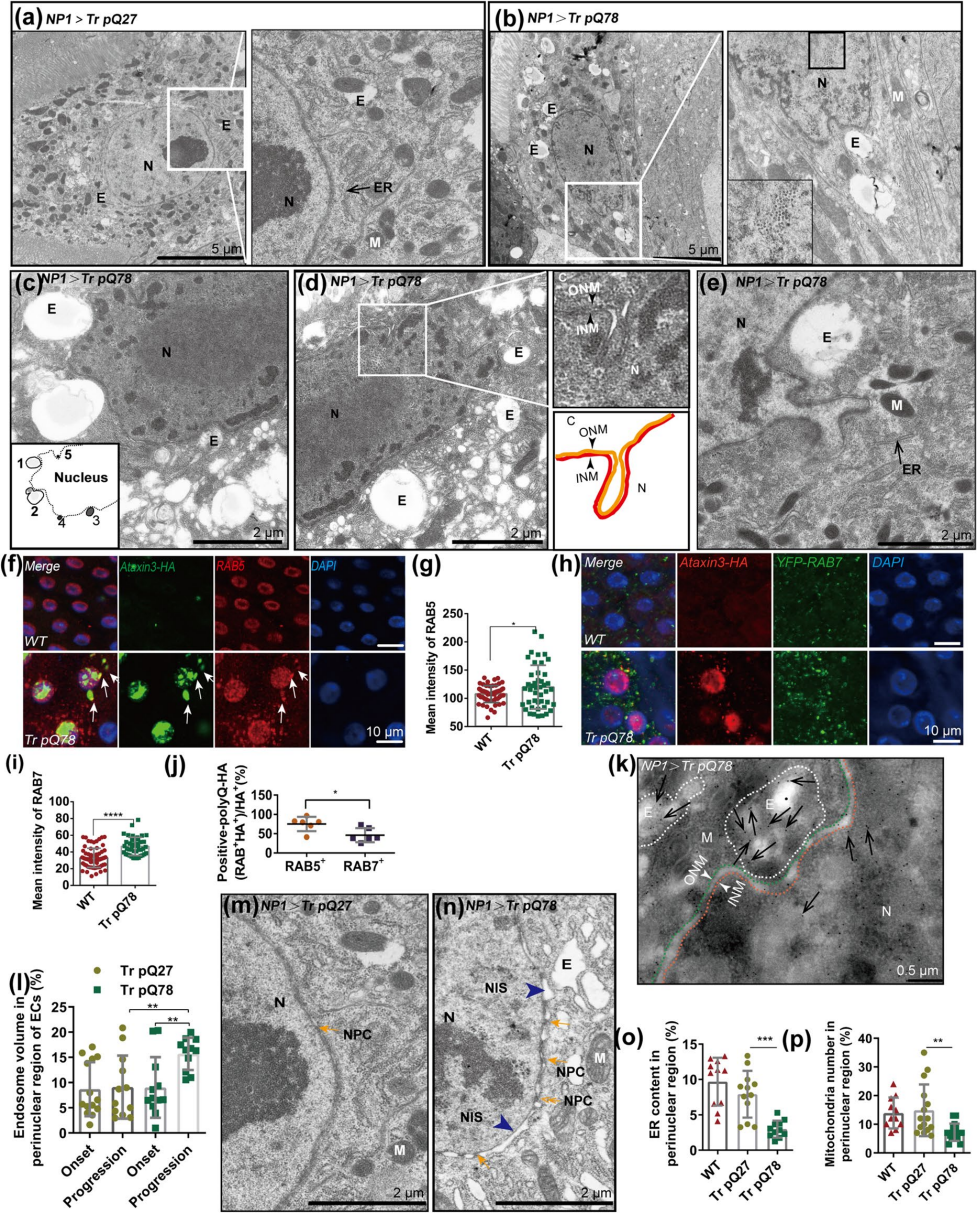
Truncated polyQ enriched in nuclear-associated endosomes with aberrant endomembrane organization **a**, **b** Representative TEM of ECs of 3-d-old Drosophila with indicated genotypes. Right panel is the local (white box) amplification of left, respectively. Intranuclear aggregates (black box) were found in the trpQ78 (truncated ataxin3 pQ78)- but not trpQ27(truncated ataxin3pQ27)-expressing ECs. In the trpQ78-expressing ECs (b), the aggregates formed into clusters near the periphery of the nucleus and endosomes were associated with the double-walled nuclear membrane invaginations (NEIs); in the trpQ27-expressing group (**a**), no aggregates were found in the nucleus with much less NEIs. N = nucleus; M = mitochondria; E = endosome; ER = endoplasmic reticulum. **C** Representative TEM showing endosomes perinuclear associated endosomes (NAEs) presumably at different stages in association with NEIs. Stages were indicated by numbers 1–5. Note the fusion of two endosomes in Stage 2. **d** A representative double-walled NEI (white box, magnified and schematic diagram on the right) in a trpQ78-expressing EC. The invagination has a “neck”, presumably for pinching off or driving scission of the INM. I/ONM = inner/outer nuclear membrane. **e** A representative NAE “docking” in a double-walled NEI in a trpQ78-expressing EC. **f** Colocalization of RAB5 (red) and trpQ78-HA (green) foci both inside and outside the nucleus. Juxtanuclear RAB5 colocalized with polyQ aggregates (indicated by white arrowheads). **g** RAB5 signal was elevated in trpQ78-expressing ECs. **h** Co-localization of YFP-RAB7 (green) with perinuclear polyQ aggregates (red) but not in the nucleus. i RAB7 signal was elevated in trpQ78-expressing ECs. **j** Immunofluorescence (RAB5 and TrpQ78-HA) and endogenous fluorescence (RAB7) data showed that polyQ78 had significantly more co-localization with RAB5 (~ 75%) than with RAB7(~ 35%). k TrpQ78-HA expressing ECs stained with gold-labelled HA for TEM. TrpQ78 signals (indicated by black arrowheads) were present in the perinuclear endosomes. l Compared with the onset of the disease (< 24 h after eclosion), endosome volume in the perinuclear region within a 2-μm radius of the outer nuclear membrane (ONM) of trpQ78-expressing ECs increased with disease progression (3–5 d old adults undergo rapid disease progression, so this stage is defined as the disease progression). **m** Normal number of NPCs and normal NIS in a trpQ27-expressing EC. NIS = nuclear intermembrane space; NPC = nuclear pore complex. **n** Loose NIS (blue arrowheads) and sparse NPCs (yellow arrows) in a trpQ78-expressing EC. **o** Reduced ER content in the perinuclear region within a 2-μm radius of the ONM of trpQ78-expressing ECs. **p** Reduced mitochondria number in the perinuclear region within a 2-μm radius of the ONM of trpQ78-expressing ECs. For immunostaining quantifications, 3 ~ 5 intestines were randomly chosen from each group, and 3 fields were randomly selected for each intestine. For TEM quantifications, at least 5 cells from different intestines were randomly chosen from each group. All values represent mean ± SD. *p < 0.05, **p < 0.01, ***p < 0.001, ****p < 0.0001 by Student’s t test (g, i, j) and one-way ANOVA (l, o, p). See also Supplementary Figure S1

We next proceeded to assess the phenotypes by transmission electron microscopy (TEM). There were no NAAs formation in the trpQ27-expressing group and the nuclear envelope presented in a smooth state with few nuclear envelope invaginations (NEIs) and no “docking” vesicles (Fig. 2a). Interestingly, in the trpQ78 group, the NAAs were unevenly dispersed and formed into clusters near the periphery of the nucleus (Fig. 2b). Numerous endosomes, which have also been termed NAEs (nuclear-associated endosomes) in previous studies on tumors (Rappa et al. 2017), were found to be associated with multiple double-walled NEIs. In the perinuclear region, the NEI and the “docking” endosomes formed mosaic structures. We found that NAEs in trpQ78 group varied in size and electron density, presumably at different stages. Some NEIs displayed a “neck”, typical structure for pinching off or driving the scission of the inner nuclear membrane (INM) (Fig. 2c–e).

In addition, we used immunofluorescence to examine the co-localization of early endosomes (EEs) or late endosomes (LEs) and trpQ78-related NAAs in the perinuclear/juxtanuclear region of ECs. The Rab5 (a previously proposed EE marker) was nearly perfectly colocalized with NAAs both in and around the nucleus (Fig. 2f, g), while RAB7-YFP (a proposed LE marker) signal tended to form perinuclear colocalization (Fig. 2h, i). Furthermore, trpQ78 seemed to cause an increase of RAB5 both in the nuclear plasma, envelope, and perinuclear region when compared to the background control or trpQ27 (Fig. 2f, Supplementary Fig.S1a–f). Tri-colored labelling demonstrated that although there was a pronounced perinuclear overlapping between trpQ78-positive RAB5 and RAB7, trpQ78 was preferentially colocalized with RAB5 when compared to RAB7 (~ 75% vs ~ 35%) (Fig. 2j). Immuno-gold TEM further confirmed the presence of trpQ78 in the endosomes close to the nuclear membrane (NM) (Fig. 2k; Supplementary Fig.S1g–i). Considering that subdomains of the NAEs have been shown to constitute a sub-nuclear compartment where biomaterials from endosomes are delivered (Rappa et al. 2017), we postulate that such “docking” endosomes in the NEI might represent a nucleocytoplasmic trafficking route for trpQ78 which needs further research.

### Aberrant endomembrane allocation caused by truncated polyQ

We noticed that different forms of endosomes accompanied the disease progression in trpQ78-expressing ECs by TEM. At the onset of the disease (24-h old adults), extensive small tubular or spherical endosomes were generated and widely distributed in the cytoplasm of ECs (Supplementary Fig.S1j); with the disease progression and the emergence of visible pathology (3–5 d old adults), endosomes in the majority of ECs were enlarged and accumulated in the juxtanuclear region, with a large number of multivesicular bodies (MVBs, Supplementary Fig.S1k). The endosomal volume and the number of MVBs in the juxtanuclear region of trpQ78-expressing group was significantly higher than that in the trpQ27-expressing group (Fig. 2l, Supplementary Fig.S1l), which was in line with the high level of the marker RAB7-YFP previously indicated by confocal immunofluorescence (Fig. 2h, i). In addition to extensive endosome accumulation and nuclear envelope invaginations (NEIs), expansion of nuclear intermembrane space (NIS) and sparse scattering of nuclear pore complexes on the NM were also observed in the trpQ78- but not trpQ27-expressing ECs (Fig. 2m, n). Compared with background control and trpQ27-expressing ECs, other membrane organelles such as ER and mitochondria were barely detected in the trpQ78-expressing group at the later disease progression stage (Fig. 2o, p, Supplementary Fig.S1m), implicating severely distorted endomembrane allocation and impaired endomembrane organization.

### RAB5 but not RAB7 participated in nuclear positioning and aggregation of trpQ78

**Fig. 3:**
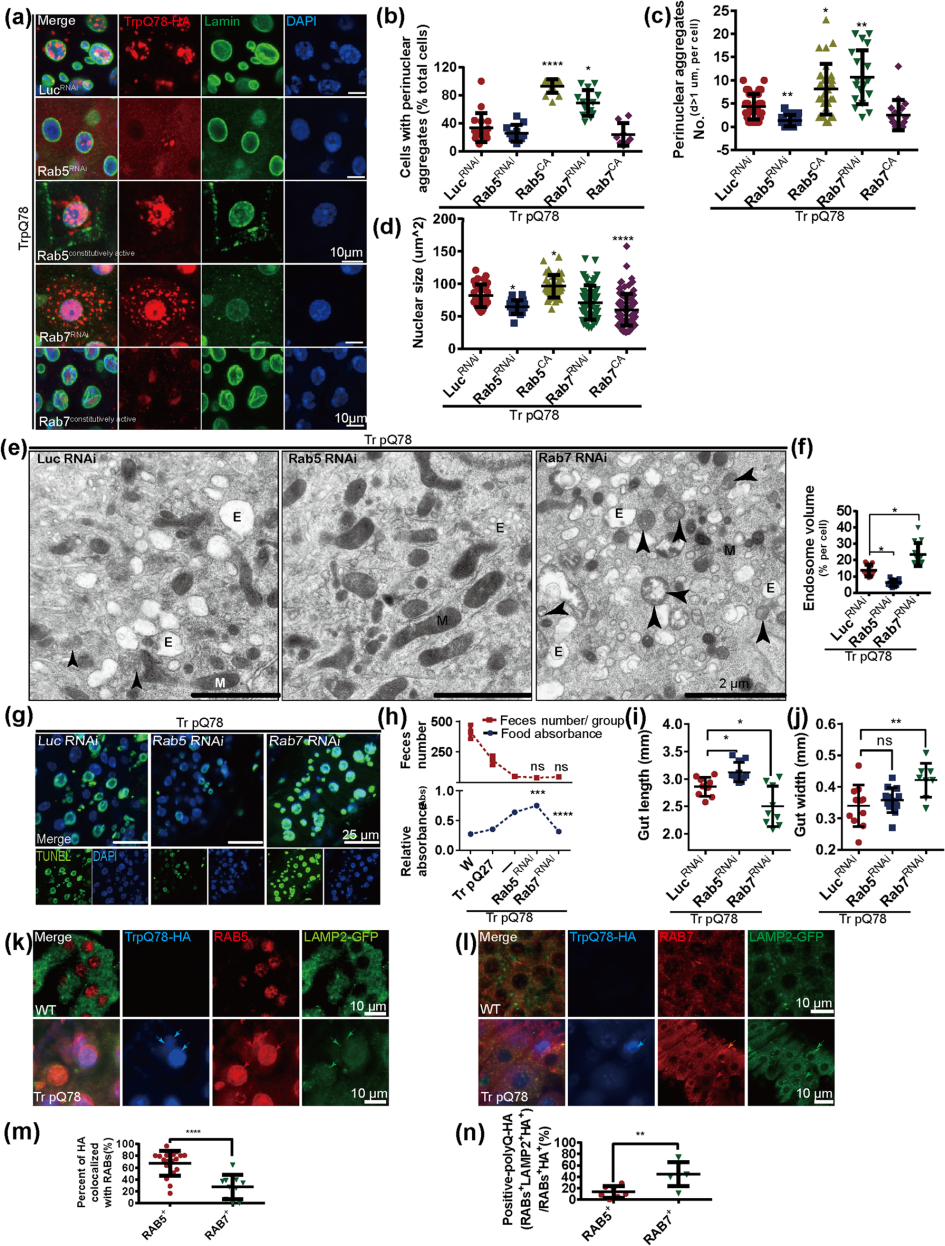
RAB5 is required for nuclear positioning and aggregation of trpQ78 **a** TrpQ78 (truncated ataxin3pQ78) and nuclear outlines in enterocytes (ECs) with the indicated genotypes were visualized by anti-HA (red), anti-Lamin (green) immunofluorescence staining. Lamin labeled the nuclear lamina and nuclei were visualized by DAPI staining. 4 ~ 5 intestines were randomly chosen from each group, and 3 fields of view were randomly selected for each intestine. **b** Percentages of cells with perinuclear aggregates increased in Rab5^CA^ or *Rab7* RNAi groups in the context of trpQ78. The perinuclear area refers to the region where the vertical distance from the nuclear membrane is the smallest radius of the nucleus. **c** *Rab5* RNAi decreased while *Rab7* RNAi increased the number perinuclear aggregates in the context of trpQ78. ECs containing at least one aggregate in (**A**) were selected for statistics. **d** *Rab5* RNAi or Rab7^CA^ decreased while Rab5^CA^ increased the nuclear size of trpQ78-expressing ECs in the context of trpQ78. **e** Representative TEM of ECs in the trpQ78-expressing group with *Luc (Luciferase)*, *Rab7*, or *Rab5* RNAi. In representative TEM fields, a large number of endosomes were observed in the *Rab7* RNAi group but rarely seen in the *Rab5* RNAi group. At least 5 cells from different intestines were randomly chosen from each group. **f** *Rab5* RNAi decreased while *Rab7* RNAi increased the polyQ-related endosome volume in comparison to *Luc* RNAi in (**e**). **g** Elevated TUNEL apoptosis signal in *Rab7* RNAi ECs in the context of trpQ78. Nuclei were stained with DAPI. 4 ~ 5 intestines were randomly chosen from each group, and 3 fields of view were randomly selected for each intestine. **h** Feeding and defecation capacity of trpQ78-expressing *Drosophila* with the indicated genetic manipulations. With *Rab7* RNAi, the intestinal excretion capacity of trpQ78-expressing *Drosophila* further decreased. Three groups with 20 flies per group were evaluated. **i, j** Length and width of guts from trpQ78-expressing *Drosophila* with the indicated genetic manipulations. *Rab7* RNAi decreased the gut length and increased the gut width in the context of trpQ78, while *Rab5* RNAi slightly increased the gut length. n ≥ 8. **k** Representative confocal image of LAMP2-YFP (green), RAB5 (red) and trpQ78-HA (blue) in the ECs. Arrows point the signals. **l** Representative confocal image of LAMP2-YFP (green), RAB7 (red) and trpQ78-HA (blue) in the ECs. Arrows point the signals. **m** The percentages of trpQ78-HA colocalized with RAB5 (~ 70%) were much higher than that with RAB7 (~ 30%), consistent with Fig. 2K. **n** Low LAMP2 positive TrpQ78^+^ RAB5^+^ ratio (~ 12%) in comparison to a much higher LAMP2 positive TrpQ78^+^ RAB7.^+^ ratio (~ 45%). All values represent mean ± SD. *p < 0.05, **p < 0.01, ***p < 0.001, ****p < 0.0001 by One-way ANOVA (**b–d, f, h–j**) and t test (**m, n**). See also Supplementary Figure S2

In previous studies, RAB5 and RAB7 were confirmed to be closely correlated with developing, turnover and degradation of endosomes (Vanlandingham and Ceresa 2009). Based on these findings, to explore the potential function of RABs on the aggregation of trpQ78-related NAAs or NAEs in intestinal ECs, we used RNAi or constitutively active (CA) to down or up-regulate their activities (Fig. 3a, Supplementary Fig.S2a–c). Different from *Rab7* RNAi, *Rab5* RNAi group exhibited decreased NAAs both intra- and peri-nuclear (Fig. 3b, c, Supplementary Fig.S2d, e). Conversely, overexpression of *Rab5* CA or *Rab7* CA demonstrated the expected opposite effects to those of RNAi manipulations (Fig. 3b, c). Furthermore, *Rabs* genetic manipulation, either *Rab5* RNAi or *Rab7* CA, also modified the enlarged nuclear size phenotypes caused by trpQ78 (Fig. 3d, Supplementary Fig.S2f, g).

Through TEM of disease groups, irregular endosomes with higher volume were observed frequently in the *Rab7* RNAi group but rarely detected in the *Rab5* RNAi group (Fig. 3e, f). It was notable that in genetic control background fruit flies, *Rab7* RNAi also resulted in an increase in the endosome volume of ECs (Supplementary Fig.S2h, i), indicating an additive effect. Furthermore, TUNEL assays showed that *Rab5* RNAi decreased the apoptotic rate in the trpQ78 background (Fig. 3g, Supplementary Fig.S2j), but not in background control group (Supplementary Fig.S2k). *Rab5* RNAi increased the feeding abilities while defecation was not altered (Fig. 3h), as were accompanied by the alterations of gut morphology (Fig. 3i, j, Supplementary Fig.S2l–n), suggesting their modifying effects on intestinal obstruction.

### Lysosomes may not be the primary transport destination for overloaded RAB5-related NAAs

We then used a reporter line, LAMP2-YFP, to label lysosome and found that the LAMP2-YFP in the trpQ78 pathological group tended to be predominantly distributed around the nucleus and exhibited remarkably low levels (Supplementary Fig.S2o–p), and the percentages of trpQ78 localized in the LAMP2 positive vesicles were rather limited (~ 15%, Supplementary Fig.S2q), suggesting the aberrant lysosomal activity mediated by trpQ78, which was in line with a previous report (Monaco and Fraldi 2020). Furthermore, through tri-colored labeling and colocalization analysis (Fig. 3k–n), we found that among total trpQ78 positive signals, there were significantly much more RAB5 positive signals (polyQ^+^ RAB5^+^, ~ 70%) than RAB7 ones (polyQ^+^ RAB7^+^, ~ 30%) (Fig. 3m), consistent with previous results (Fig. 2j, k). More importantly, among the total polyQ^+^ RAB5^+^ signals, only a small fraction was LAMP2 positive (~ 12%), while among the total polyQ^+^ Rab7^+^ signals, the LAMP2 positive fraction was much higher (~ 45%) (Fig. 3n), indicating different predominant routes for RAB5^+^ trpQ78 and RAB7^+^ trpQ78, respectively. Lysosomes may not be the primary transport destination for overloaded RAB5-related NAAs. While those NAAs in the RAB7 positive vesicles, are more prone to LAMP2^+^ vesicle-mediated processing. These divergent trafficking routes seemed to be delicately orchestrated as we also noticed that *Rab7* RNAi enhanced RAB5-positive NAAs around the nucleus, although the expression level of RAB5 was not significantly affected (Supplementary Fig.S2b, c). How Rab5 and Rab7 are coordinated in this pathological context needs further study. In addition, although RABs, especially RAB5, had extensive co-localization with NAAs, an interaction between RAB5 and trpQ78 was not detected with the immunoprecipitation technique (Supplementary Fig.S2r), and the molecular mechanisms remain to be disclosed in the future.

### Unique distribution of Atgs accompanied with nuclear-associated polyQ aggregates formation

**Fig. 4:**
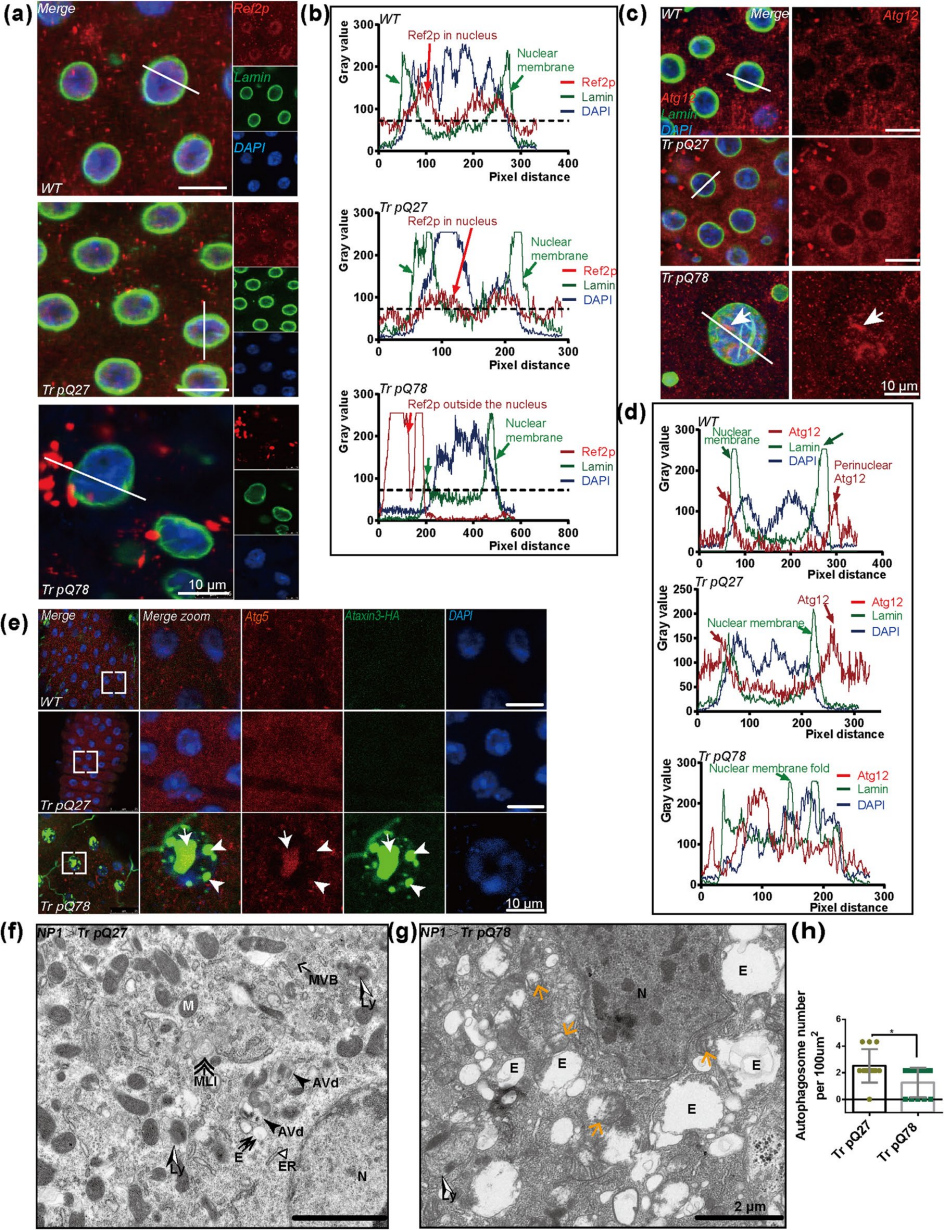
Atgs with altered expression profiles were enriched in nuclear-associated polyQ aggregates **a** Ref2p mainly localized in the nucleus with small puncta outside the nucleus in the wild-type genetic background control and trpQ27 (truncated ataxin3pQ27)-expressing groups but formed large aggregates outside the nucleus in trpQ78 (truncated ataxin3pQ78)-expressing group. Ref2p and nuclear membrane were stained with anti-Ref2p (red) and anti-Lamin (green), respectively. Nuclei were marked by DAPI. **b** Densitogram analysis of the selected region of interest (ROI, white line) in control and polyQ-expressing groups in (A). **c, d** Representative image and corresponding densitogram analysis of ROI showed Atg12 located inside the nucleus in trpQ78-expressing group but mainly distributed in the perinuclear region in the control and trpQ27 expressing groups. Atg12 and nuclear membrane were stained with anti-Atg12 (red) and anti-Lamin (green), respectively. **e** Co-localization of Atg5 and trpQ78-HA aggregates. Atg5 and trpQ78 were stained with anti-Atg5 (red) and anti-HA (green) antibodies, respectively. Nuclei were visualized by DAPI staining. **f** Degrading autophagic vacuoles (AVds), classic multiple vesicular bodies (MVBs), lysosomes (Lys), and multilamellar inclusions (MLIs) in the perinuclear region of a trpQ27-expressing EC, demonstrated by TEM. N = nucleus. **g** The endosomes (E) of different sizes densely distributed, and some of them were filled with a large amount of content (yellow arrows). Autophagosomes and lysosomes were rare in the trpQ78-expressing ECs, demonstrated by TEM. **h** Autophagosome number decreased in trpQ78-expressing ECs. *p < 0.05 by Mann–Whitney test. All values represent mean ± SD. For immunostaining test, at least 3 intestines were randomly chosen from each group, and 3 fields of view were randomly selected for each intestine. For TEM test, at least 5 cells from different intestines were randomly chosen from each group. See also Supplementary Figure S3

Autophagy pathway disturbance has been reported as a hallmark of polyglutamine diseases (Kegel et al. 2000; Rui et al. 2015). To this end, we detected higher mRNA levels of *Atg* factors in the ECs of the trpQ78 model than those of the background control and the trpQ27-expressing group (Supplementary Fig.S3a). We then examined the distribution of these Atgs with immunostaining of Ref2p (The homologous gene of *p62* in *Drosophila* is *ref2p*), Atg12, Atg5, and Atg8/LC3 (Fig. 4a–e, Supplementary Fig.S3b–d). The distribution of Ref2p was unique: in the trpQ27-expression group, similar to that in wild-type background group, Ref2p mainly distributed in the nucleus with small puncta scattering in the cytoplasm (Fig. 4a, b, Supplementary Fig.S3b); in the trpQ78 group, Ref2p formed larger puncta in the cytoplasm with very little nuclear localization. In the background control group, Atg12 mainly distributed around the nucleus, and under pathological conditions, the protein signals were co-localized with trpQ78 aggregates (Fig. 4c, d, Supplementary Fig.S3c). A small amount of distribution of Atg8/LC3, Atg5, and Ubiquitin were also detected in the trpQ78 aggregates (Fig. 4e, Supplementary Fig.S3d, e). Interestingly, in comparison to the background control ECs in which there were abundant autophagosomes, lysosomes, and multilamellar inclusions, few autophagosomes and lysosomes were found in trpQ78-expressing ECs under TEM (Fig. 4f–h), which was in agreement with the previous results of LAMP2-YFP labeling experiment (Supplementary Fig.S2o–q) and indicated that either a deficiency in the autophagic capacity or possible non-canonical autophagy-related roles exerted by ATGs.

### Atgs gene manipulation differentially influenced disease severity

**Fig. 5:**
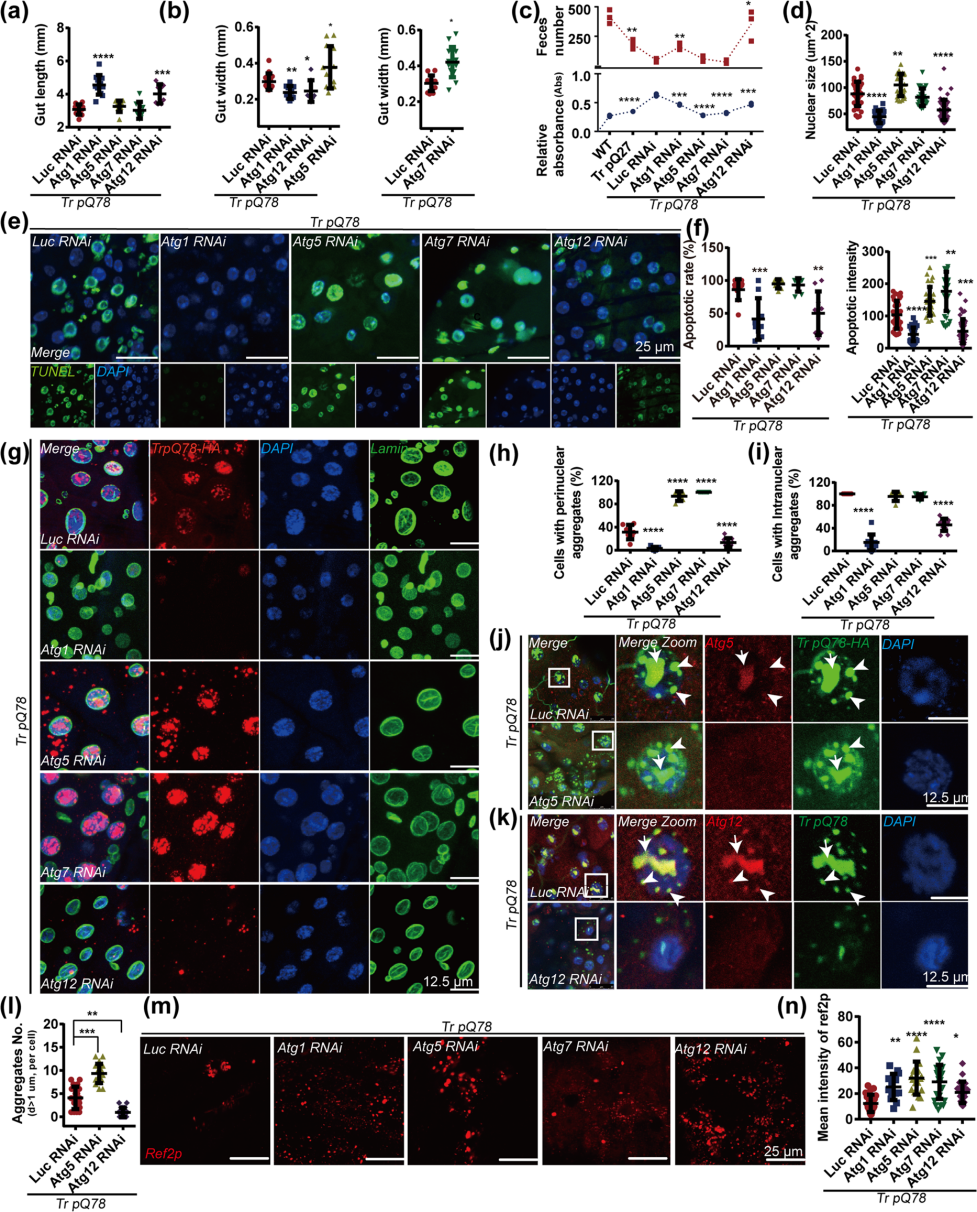
*Atgs* gene-manipulation differentially modulated the nuclear-associated aggregation and toxicity of polyQ **a, b** *Atg1* and *Atg12* down-regulation restored the gut length and reduced gut-swelling while *Atg5* RNAi aggravated gut swelling in the context of trpQ78 (truncated ataxin3pQ78) (*n* ≥ 8). **c** Feeding and defecation capacity of flies with *Luc* (*Luciferase)* RNAi or *Atgs* RNAi in the context of trpQ78. *Atg1* and *Atg12* RNAi restored, while other *Atgs* RNAi suppressed food intake with very low food absorbance and feces discharge in trpQ78 flies (20 *Drosophila*/group, 3 groups/genotype). **d** *Atg5* RNAi further increased while *Atg1* or *Atg12* RNAi reduced the nuclear size significantly in the context of trpQ78. **e, f** TUNEL analysis showed that less apoptosis was induced in the guts with *Atg1* RNAi or *Atg12* RNAi but not with *Atg7* RNAi or *Atg5* RNAi in the context of trpQ78. *Atg5* RNAi and *Atg7* RNAi increased while *Atg1* RNAi and *Atg12* RNAi decreased the apoptotic intensity of trpQ78-exressing ECs. Apoptotic rate and signal intensity were quantified, respectively. Nuclei were stained with DAPI. **g** TrpQ78 and nuclear membrane outlines in ECs with the indicated genotypes were visualized by anti-HA (red), anti-Lamin (green) immunofluorescence staining. Nuclei were visualized by DAPI staining. **h** Percentages of ECs with perinuclear aggregates decreased in *Atg1* or *Atg12* RNAi groups but increased in *Atg5* and *Atg7* RNAi groups in (**g**). The perinuclear area referred to the region where the vertical distance from the nuclear membrane is the smallest radius of the nucleus. **i** Percentages of ECs with intranuclear aggregates decreased in *Atg1* or *Atg12* RNAi groups in (**g**). **j** *Atg5* RNAi increased the accumulation of trpQ78 aggregates (green) both in juxtanuclear and intranuclear regions. ATG5 signal (red) was reduced after RNAi. Nuclei were visualized by DAPI staining. **k** TrpQ78 puncta (green) in *Atg12* RNAi group were less and distributed far away from nucleus. ATG12 signal (red) was reduced after RNAi. **l** Quantifications of polyQ aggregates in (**j**) and (**k**). **m** Ref2p patterns in ECs with indicated genotypes were visualized by anti-Ref2p (red) immunofluorescence staining. Nuclei were visualized by DAPI staining. **n** Higher levels of Ref2p signal with all *Atg* RNAi manipulations in the context of trpQ78. All values represent mean ± SD. *p < 0.05, **p < 0.01, ***p < 0.001, **** *p* < 0.0001 by One-way ANOVA. For immunostaining test, 3 ~ 5 intestines were randomly chosen from each group, and 3 fields of view were randomly selected for each intestine. For TEM test, at least 5 cells from different intestines were randomly chosen from each group. See also Supplementary Figure S4

We then used RNAi to examine the involving function of ATGs and again independent RNAi lines were used where applicable. *Atgs* gene manipulation alone had no significant influence on the gut morphology of background control *Drosophila* except minor impact by *Atg1* RNAi (Supplementary Fig.S4a). Remarkably, down-regulation of *Atg1* and *Atg12* had a restorative effect on the shortened and deformed intestine of the trpQ78 flies, while down-regulation of *Atg5 o*r *Atg7* worsen the distortion (Fig. 5a, b, Supplementary Fig.S4b). As described above, trpQ78 *Drosophila* displayed severe food retention with little excretion, a manifestation of severe intestinal obstruction. Down-regulation of *Atg1* or *Atg12* promoted intestinal excretion and mitigated the intestinal obstruction, whereas down-regulation of *Atg5*, or *Atg7* hindered the activities of eating or defecating (Fig. 5c). In *Atg1* or *Atg12* RNAi groups, the restored gut morphology coincided with a decrease of the nuclear size in ECs (Fig. 5d, Supplementary Fig.S4c–e). When Atgs were down-regulated in the background control context, only *Atg1* RNAi caused a slight change in nuclear size (Supplementary Fig.S4f). Consistently, the groups with severe intestinal obstruction in the context of trpQ78 (that is, *Atg5*, *Atg7* RNAi) displayed more severe apoptosis, while the *Atg1* and *Atg12* RNAi groups, which had mitigated intestinal obstruction, had reduced apoptosis levels (Fig. 5e, f). Unlike the pathological groups, in the background control group, *Atgs* gene manipulation did not significantly affect the level of intestinal apoptosis (Supplementary Fig.S4g).

### Atgs RNAi differentially influenced polyQ aggregates formation and distribution

*Atg5* or *Atg7* RNAi reduced the percentages of ECs with trpQ78 aggregates, while *Atg1* or *Atg12* RNAi had the opposite effect (Fig. 5g–i, Supplementary Fig.S4h–j). Interestingly, when Atg12 was down-regulated, trpQ78 aggregates were distributed in the periphery of cells. Together, these results conclude that trpQ78 aggregate alterations differentially induced by *Atgs* RNAi are closely associated with disease severity. Given the functions of ATG5-ATG12 are deemed to be consistent in the canonical autophagy pathway (Romanov et al. 2012), we examined the involvement of ATG5-ATG12 more thoroughly. Downregulation of them individually decreased the formation of ATG5-ATG12 complex as expected (Supplementary Fig.S4k), but had opposite effects on the formation and distribution of trpQ78 aggregates (Fig. 5j–l), indicating that ATG5-ATG12 complex might not be the primary contributing factor in this context while ATG5 and ATG12 alone might separately exert non-canonical functions.

Furthermore, the levels of Ref2p, which usually inversely correlate with autophagic flux, were surprisingly up-regulated in all of the *Atgs* RNAi treatments despite their differential modifying effects (Fig. 5m, n), once again indicating that non-canonical autophagy pathway was involved although it remained unclear why Ref2p increased and aggregated in the cytoplasm after *Atgs* down-regulation, which derived further investigation. Collectively, based on all the aforementioned results, we propose that the non-canonical autophagy pathway of ATGs modulates the trpQ78 toxicity in this disease model.

### Non-canonical roles of Atgs differentially affected the size and distribution of RAB5-positive NAAs and trpQ78-related NAEs

**Fig. 6:**
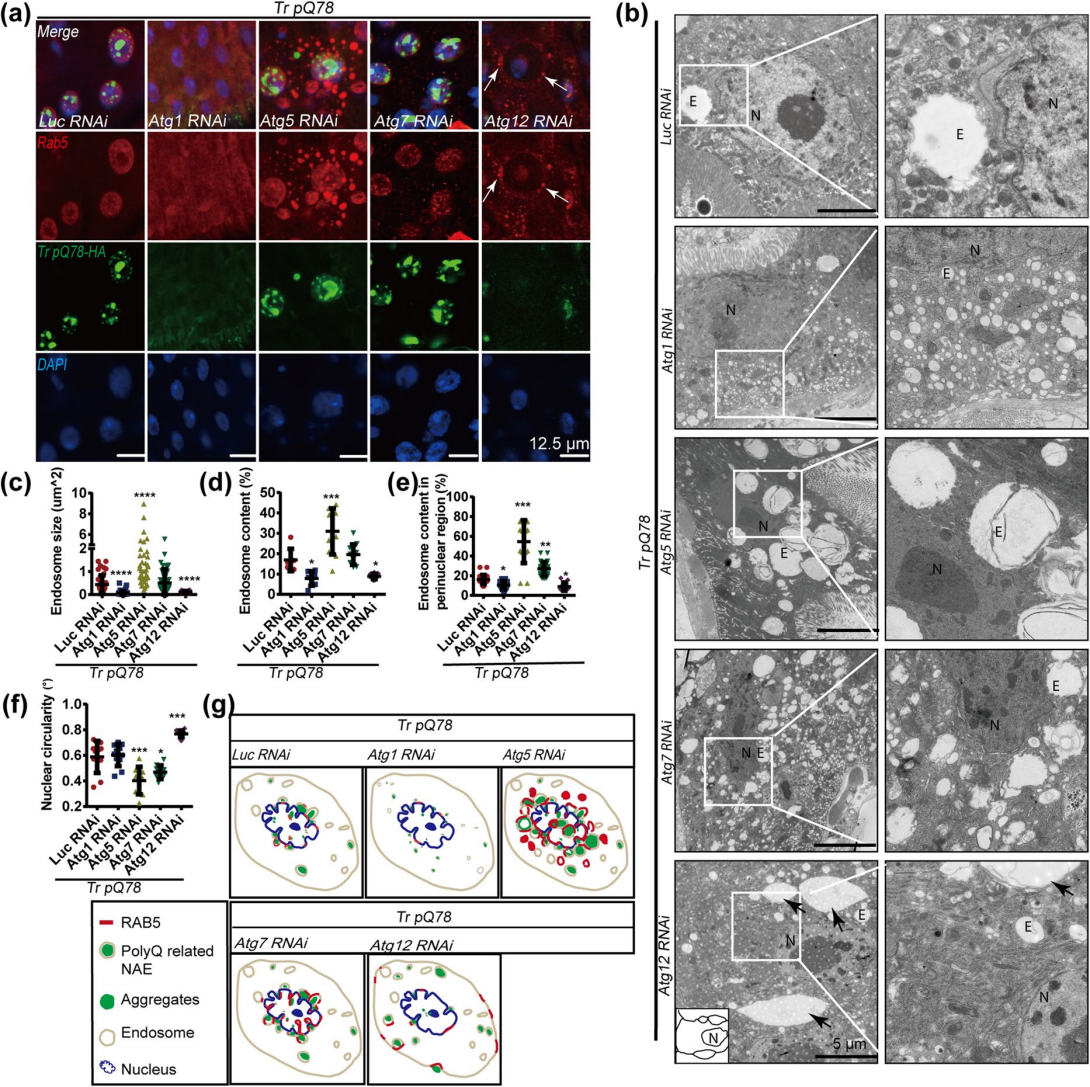
Non-canonical roles of ATGs differentially affected the size and distribution of nuclear-associated endosomes **a** RAB5 and trpQ78 (truncated ataxin3pQ78) were coincidently influenced by different *Atgs* genetic manipulations. RAB5 (red) and trpQ78-HA (green). Nuclei were visualized by DAPI staining, *n* ≥ 4. Arrows indicated possible intercellular RAB5 signals. **b** Representative TEM of trpQ78-expressing ECs with control (*Luciferase, Luc*) or *Atgs* RNAi. Arrows indicated the intercellular endosome-like structures. Scale bars in first column: 5 μm. The picture in the second column corresponds to a partial enlargement of the first column. **c** *Atg1* or *Atg12* RNAi decreased while *Atg5* RNAi increased the endosome size in the context of trpQ78. **d** *Atg1* or *Atg12* RNAi decreased while *Atg5* increased the endosome content in the context of trpQ78. **e** *Atg1* or *Atg12* RNAi decreased while *Atg5* or *Atg7* RNAi increased the endosome content in the perinuclear region (2-μm radius of the nuclear membrane) in the context of trpQ78. **f** The nuclei of the *Atg5* RNAi group were more irregular, while those of *Atg12* RNAi were rounder than those of the *Luc* RNAi group in the context of trpQ78. **g** Schematic diagram of endosome and trpQ78 aggregate distribution in ECs of flies with indicated genotypes. All values represent mean ± SD. *p < 0.05, **p < 0.01, ***p < 0.001, **** *p* < 0.0001 by Kruskal–Wallis test (**c**), and by One-way ANOVA (**d–f**). For immunostaining test, at least 5 intestines were randomly chosen from each group, and 3 fields of view were randomly selected for each intestine. For TEM test, at least 5 cells from different intestines were randomly chosen from each group. See also Supplementary Figure S5

Immunostaining further demonstrated that RAB5 and trpQ78 were concordantly influenced by different *Atgs* genetic manipulations. *Atg5* and *Atg7* RNAi induced stronger RAB5 signal and trpQ78 aggregates both in and around the nucleus of ECs, with much more intensifying effect for *Atg5* RNAi, even in the genetic background control (Fig. 6a, Supplementary Fig.S5a). In contrast, when *Atg1* or *Atg12* was down-regulated, trpQ78 and RAB5 co-accumulation were barely detected either in or around the nucleus. More interestingly, down-regulating *Atg12* led to RAB5 aggregation at the periphery of cells (Fig. 6a, Supplementary Fig.S5a).

TEM results demonstrated that ATGs significantly affected the size and distribution of endosomes in the context of trpQ78 overexpression (Fig. 6b). Compared with the endosomes of the control RNAi group, the endosomes of the *Atg1 o*r *Atg12* RNAi ECs were dispersed in the cytoplasm; in contrast, in the *Atg5* or *Atg7* downregulation groups, endosomes accumulated more around the nucleus (Fig. 6b). Furthermore, *Atg1* or *Atg12* RNAi displayed reduced endosomal sizes, whereas the *Atg5* RNAi group harbored enlarged endosomes (Fig. 6a, c, note that in the *Atg12* knock-down group, the extra-large intercellular endosome-like structures, which were consistent with the observation of possible intercellular RAB5 aggregation observed in the immunofluorescence, were not taken into account). The volume of the endosome increased significantly after *Atg5* but decreased after *Atg1* or *Atg12* RNAi in the trpQ78 context (Fig. 6d). To better analyze the distribution of NAEs, we measured the volume of the endosomes within a radius of 2-μm from the nuclear membrane (NM). The endosomal volume in this region decreased significantly when *Atg1* or *Atg12* was down-regulated, but increased in *Atg5* or *Atg7* RNAi group (Fig. 6e). Interestingly, in the Atg12 RNAi group, the otherwise wrinkled and curly NM in trpQ78 became smooth (Fig. 6f). In parallel, *Atg5* and *Atg7* RNAi had little effect on the endomembrane system of background control flies (Supplementary Fig.S5b–d). We also examined the effect of *Atg8a* RNAi on trpQ78, which seemed to be more complicated and the mechanisms need to be studied further.

Collectively, these results indicated that the size, volume, primary deposit site, distribution and trafficking of trpQ78-related NAEs could be differentially modulated by ATGs. The overall impacts or ATGs on trpQ78 were summarized (Fig. 6g), which implicated that ATGs might differentially modify the trpQ78-related pathologies through non-canonical roles. Inspired by previous studies on amphisome-like vesicles, a category of RABs-, and ATGs-positive single membrane organelle for non-degradable cargos and signaling factors trafficking independent of canonical autophagy (Han et al. 2023; Sanchez-Wandelmer and Reggiori 2013), we speculate that such polyQ-related endosomes found in our study can be an amphisome-like structure with transport functions.

## Discussion

In summary, we established the first polyQ-mediated intestinal obstruction model by ectopical expression of polyQ expanded ataxin3 in the *Drosophila* intestinal ECs. In the damaged ECs, enhanced polyQ toxicity was correlated with excessive accumulation of juxtanuclear polyQ aggregates, which were present in perinuclear associated endosomes (NAEs), indicating an impaired nuclear internalization route mediated by endosomes. In addition to an over-active nuclear membrane, the endomembrane system was severely disorganized: endosomes or endosome derivatives excessively accumulated. Our genetic and cellular analyses further demonstrated that endocytic pathway-related factors (RABs), autophagy machinery (Atgs) were all involved in regulating polyQ-related NAEs. Notably, Atgs appeared to exhibit non-canonical autophagic roles in this response. Taken together, our findings underscored the important role of endosome-mediated nucleocytoplasmic trafficking and homeostatic endomembrane allocation in the pathogenesis of polyQ.

Many neurodegenerative diseases including SCA3 are systemic diseases. The pathological protein is widely expressed in the gastrointestinal tract, renal epithelium, fibroblasts, lymphocytes and even cardiomyocytes, and produces a broad spectrum of clinical symptoms (Bradford et al. 2010; Moffitt et al. 2009; Schuster et al. 2022). In fact, in neuronal inclusion diseases, the viscera system is equally vulnerable, and, like in the neurons, pathological protein inclusions or aggregates can easily form in the visceral epithelium. Furthermore, the epithelial cells in the intestine and skin are relatively large and can be used as an alternative of neurons to study the mechanisms of polyQ and other poly expansion diseases.

Given the heterogeneity of polyQ proteins, the form, fraction, and distribution and toxicity of polyQ within cells can be diverse (Leitman et al. 2013; Lu et al. 2019). We noticed that in the trpQ78-expressing ECs, polyQ aggregation was specially enriched in nucleus, intra- or peri-nuclear regions, formed into clusters, and unevenly dispersed. This kind of aggregated particles formed by truncated polyQ (with the removal of N-terminal fragments) is more toxic than those uniformly dispersed (Haacke et al. 2006; Lunkes et al. 2002). The overlapping between trpQ78 and endosomes provides a new perspective of trpQ78 toxicity produced by endomembrane disturbance (Fig. 6a). Recent research showed that on the outside of the nuclear membrane, there is a complex and finely assembled trilayer construction including RAB5-positive endosomes, whose function is to retrograde endocytic trafficking to regulate plasma membrane growth and secretion of basement membrane proteins (Zheng et al. 2020). Our research opened up a new perspective, explaining that the function of these endosomes was not limited to the cytoplasm, but went deep into the nuclear regions, forming tunnel-like transportation pipelines, and participating in the transportation of substances into the nucleus.

Sustainable and reversible recycling of endosomes is important for the homeostasis of the endomembrane system (e.g. ER, TGN, lysosome, endosome, autophagosome, amphisome, NM, and plasma membrane) (Bonifacino and Glick 2004). In the perinuclear region of trpQ78-expressing ECs, in sharp contrast to the excessive accumulation of endosomes and NM at the disease progression stage, no increase of autophagosomes and lysosomes were observed. As the disease progressed further, ER and mitochondria were significantly reduced, implicating that the endomembrane allocation and homeostasis had been disrupted, very likely due to the insatiable consumption of polyQ trafficking.

During endosomal trafficking, through iterated processes of budding, fission and fusion, then the cargo reaches its final destinations within or outside the cells (Chaumet et al. 2015; Raices and D'Angelo 2012; Rappa et al. 2017; Zheng et al. 2020). According to the difference of the destinations, these vesicle-traffickers, including early endosomes (EEs), late endosomes (LEs), lysosomes, and amphisomes, etc., can be classified into the degradative or non-degradative types, with lysosomes as the destination of the degradative type while nucleoplasm or extracellular space as the destination of the non-degradative type. It has been proposed that non-degradative trafficking route play important roles in cargo transport, signal nuclear-internalization and material exocrine (Chaumet et al. 2015; Rappa et al. 2017; Sanchez-Wandelmer and Reggiori 2013). The special type of trpQ78-positive endosomes identified in this study were very similar to the amphisome-like monolayer trafficker found in previous reports (Sanchez-Wandelmer and Reggiori 2013). As the disease progressed, these polyQ-related vesicles gradually aggregated in the juxtanuclear region. Since polyQ has irreversible damage to the autophagosome-lysosome system, the degradative pathway may have a limited effect on the clearance of polyQ-positive endosomes as the disease progresses (Wong and Holzbaur 2014). Therefore, when the endosomal degradative and non-degradative pathways are overloaded or damaged, avoiding excessive juxtanuclear aggregation by delicate manipulation of endosome trafficking activity or routes in the cytoplasm could be a potential strategy to alleviate disease progression.

On the other hand, the observation of highly-active nuclear envelope invaginations (NEIs) coinciding with the emergence of “docking in” endosomes suggested that non-degradative NAEs may initiate increased trafficking across the nuclear membrane (NM) (Supplementary Fig.S6a). RAB5-positive endosomes may carry some of the pathological proteins, gathering around and invading the nucleus. Some of these endosomes might develop into RAB7-positive ones, which are eventually degraded by the lysosomal pathway in the cytoplasm. When RAB7, which has been shown to affect the endosome-lysosome turnover pathway (Chaumet et al. 2015; Santos et al. 2018; Vanlandingham and Ceresa 2009), was down-regulated, the perinuclear polyQ-related NAEs increased and the overall pathology aggravated. Considering the low level of autophagosome-lysosome, we hypothesize that RABs might influence the NAEs juxtanuclear loading and toxicity through the endosome-mediated nucleocytoplasmic trafficking pathway. Since *Rab5* RNAi upregulates the levels of Rab7 in our research, we speculate that Rab5, through the influence on Rab7, may partially but not completely modulate pathological aggregation only in the cytoplasm. Notably, the simple downstream relationship of RAB5 and RAB7 made it unexplainably for the rarely existence of RAB7 but rich aggregation of RAB5 in the polyQ aggregates especially in the nuclear, we also noticed that RAB5 but not RAB7 exhibits a stronger correlation and colocalization with pathological deposition both perinuclear or in nucleus.

The endosomes-mediated nucleocytoplasmic trafficking route described here shares similarities with mechanisms observed in nuclear egress of ribonucleoprotein particles or nuclear ingress of herpes viruses. These mechanisms involve increased curvature of the nuclear membrane (NM) with 'docking in' endosomes (Rappa et al. 2017; Santos et al. 2018; Speese et al. 2012; Wu et al. 2014). In our study, some polyQ^+^ RAB5^+^ endosomes interfaced with the “dock-in” NM, which is very likely in a state of active segregation, immobilization and even acceptance of endosomes. As it is still unclear how cargos pass through the double-layer NM, based on the existence of “dock-in” structure, we speculate that the membrane fusion and terminal unpacking procedures may be the one of the important steps of trans-nuclear transport in these cases (Supplementary Fig.S6b), and the details of mechanism deserve further study.

**Fig. 7:**
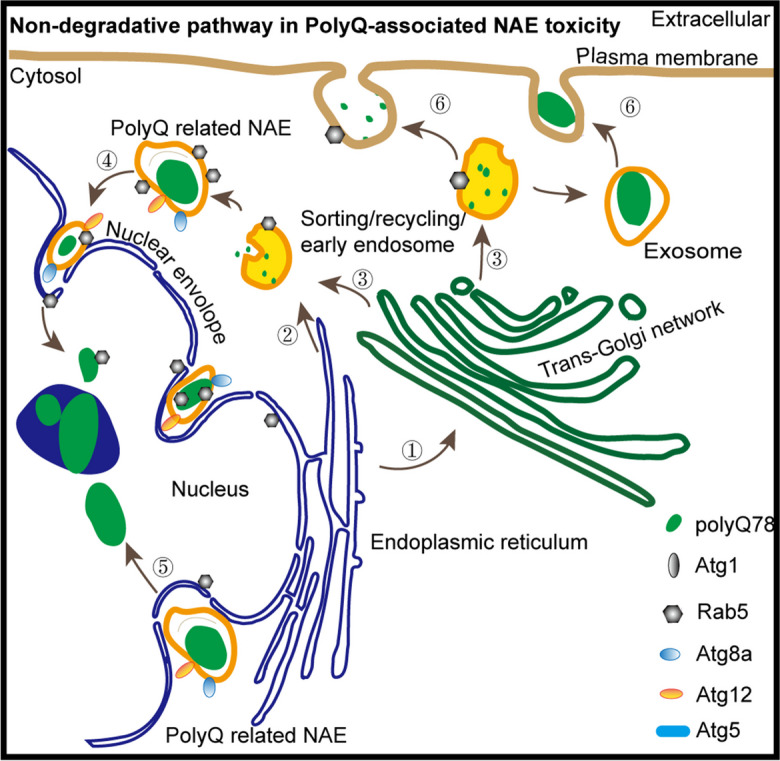
Schematic model of non-degradative pathway in PolyQ-related NAE (envelope invagination-associated endosome) toxicity. **a** Apart from the classical endosome-autophagosome-lysosome degradative pathway, traffic between different endomembrane organelles (1–6), forming non-degradative pathways that influence PolyQ burden, especially in and around the nucleus. **b** Accumulated NAE burden and endomembrane disorganization partially reflect high trpQ78-toxicity. **c** A series of modifying factors including autophagy-related proteins and Rabs systematically regulate trpQ78 nucleocytoplasmic trafficking and toxicity. See also Supplementary Figure S6. PolyQ mainly refers to trpQ78 (truncated ataxin3pQ78)

The autophagy pathway plays important roles in the quality control of cytoplasmic proteins and aggregate elimination. In the current study, Atgs were found to affect the volume, primary deposit site and trafficking of trpQ78-associated endosomes rather than canonical autophagic processes (Fig. 7, Supplementary Fig.S6c). As summarized in Supplementary Table 1 and Fig. 6g, through inhibiting *Atg1*, a key initiator of endosomal formation and development, polyQ toxicity was partially inhibited. In the canonical autophagy pathway, Atg5 and Atg12 form a complex to influence quality control of cellular proteins or organelles (Romanov et al. 2012). To our surprise, there was no increase of basic autophagic levels indicated by the Atg5-Atg12 complex in trpQ78-expressing ECs, while *Atg12* and *Atg5* down-regulation had different effects on polyQ-related aberrant endosomes and toxicity. This finding is in agreement with a previous study on tumor exosome production showing that similar to that of Atg5 overexpression, when *Atg12* was down-regulated, endosomes tended to be transported to the periphery of cytoplasm (and were eventually released extracellularly) (Guo et al. 2017). We postulate that multiple Atgs, especially Atg1, Atg5 and Atg12, determine the assembly, transport direction and turnover of polyQ-related endosomes, thereby maintaining the homeostasis of the autophagy system and endosome system.

Taken together, our findings uncover an intricate endosome-centered control system for both protein quality and endomembrane homeostatic organization, which may have an impact on the treatment of a broad spectrum of PolyQ diseases. Although there are some reports of PolyQ diseases occurring in extraneural tissues like the intestine, it's widely acknowledged that these diseases primarily affect the brain. The pathogenic mechanisms involved are complex, with different regions of the same brain potentially showing distinct pathogenicities. Whether the mechanisms observed in our study on PolyQ proteins in the intestine are applicable to the nervous system requires further investigation.

## Supplementary Information

Supplementary Material 1.

## Data Availability

No datasets were generated or analysed during the current study.

## Abbreviations

AVd: Degrading autophagic vacuoles
ECs: Enterocyte cells
EE: Early endosome
ER: Endoplasmic reticulum
I/ONM: Inner/outer nuclear membrane
LE: Late endosome
MLI: Multilamellar inclusion
MVB: Multivesicular body
NAAs: Nuclear-associated aggregates
NAE: Nuclear-associated endosome
NEI: Nuclear envelope invagination
NIS: Nuclear intermembrane space
NPC: Nuclear pore complex
NM: Nuclear membrane
PolyQ: Polyglutamine
ROI: Region of interest
SCA3/ MJD: Spinocerebellar ataxia type 3/Machado-Joseph disease
TUNEL: Terminal deoxynucleotidyl transferase-mediated dUTP-biotin nick end labeling
