# Supplementary material for: Endosome mediated nucleocytoplasmic trafficking and endomembrane allocation is crucial to polyglutamine toxicity
Source: Cell Biol Toxicol. 2024 Jun 20;40(1):48. doi: 10.1007/s10565-024-09891-4 (PMC11189978; doi:10.1007/s10565-024-09891-4)
Supplement: Supplementary file 1 — Supplementary Material 1. [file 10565_2024_9891_MOESM1_ESM.docx]

Endosome mediated nucleocytoplasmic trafficking and endomembrane allocation is crucial to polyglutamine toxicity

Running Title

Endosome mediate polyQ trafficking and toxicity

Yuyu Nan ^1，2^, Wenfeng Chen^3^, Fei Chen ^3^, Lili Wei ^4^, Aiyuan Zeng ^5,6^ , Xiaohui Lin ^5,6^, Wenbin Zhou ^1^, Yufeng Yang ^3,^* and Qinghua Li ^5,6,^*

^1^ Department of Neurology, Xiangya hospital, Central South University, Changsha, China, 410000; [tijsnyy89@163.com](mailto:tijsnyy89@163.com)；

^2^ Department of Critical Care Units, The First Affiliated Hospital, Zhejiang University School of Medicine, Hangzhou, China, 311121; [1321267@zju.edu.cn](mailto:1321267@zju.edu.cn)

^3^ Institute of Life Sciences, Fuzhou University, Fuzhou, Fujian Province, China, 350108

^4^ Guangxi Clinical Research Center for Neurological Diseases, Guilin, Guangxi, China, 541001

^5^ Department of Neurology, The Affiliated Hospital of Guilin Medical University, Guilin, Guangxi, China, 541001

^6^ Guangxi Key Laboratory of Brain and Cognitive Neuroscience, Guilin, Guangxi, China, 541004

***** Correspondence: [qhli1999@163.com](mailto:qhli1999@163.com); Tel.: +86-1587-836-1508(Qinghua Li.), [yangyf@fzu.edu.cn](mailto:yangyf@fzu.edu.cn); yangyufeng@outlook.com; Tel.: +86-1865-033-7990(Yufeng Yang)

Materials and methods

***Drosophila* strains**

Unless otherwise mentioned, flies were raised on a standard agar/cornmeal/yeast diet at 25°C until adulthood, when they were housed at 29°C. Unless otherwise mentioned, 5-d-old male animals were used in the experiments. All experiments follow single-blind test. UAS-*Atg1* RNAi (THU2357, THU5552), UAS-*Atg5* RNAi (THU2714, THU1481), UAS-*Atg7* RNAi (THU2793, THU1583), UAS-*Atg8a* RNAi (THU1555), UAS-*Atg12* RNAi (THU3667, THU2715), UAS-*Rab5* RNAi (THU0679, THU3215), UAS-*Rab7* RNAi (THU2437, THo2539.N), were obtained from Tsinghua University *Drosophila* Resource Center (Beijing, China). The UAS-*Luciferase* RNAi (31603), YFP-myc labelled RAB7 knock-in strain (62545), UAS-LAMP2-YFP (42714), UAS-RAB7-YFP (23641), UAS-RAB5 ^constitutively active^ (9773), UAS-RAB7 ^constitutively active^ (9779) and other *Drosophila* strains were obtained from Bloomington *Drosophila* Stock Center (Bloomington, IN, USA). Pathological SCA3 strains were as follows: UAS-ataxin3trpQ78 (8150) expresses a HA-tagged C-terminal fragment of the human ataxin3 with a 78-repeat polyglutamine tract; UAS-ataxin3flpQ84 (33610) expresses an N-terminally myc-tagged full length ataxin3 with an 84-repeat polyglutamine tract. SCA3 controls included UAS-ataxin3trpQ27 (8149) and UAS-ataxin3flpQ27 (33610). In accordance with the genetic background, we used w^1118^ flies as controls. Unless otherwise mentioned, over-expression of exogenous or RNA-interference of endogenous genes in *Drosophila* intestinal enterocytes (ECs) were controlled by using the *NP1*-Gal4 driver, which encompasses an enhancer trap in the intestine-specific brush border myosin IA gene. The specimens used in the immunofluorescence assay were harvested from the intestines of *Drosophila*. Whole intestines were used in immunoblotting and QPCR assays.

**Antibodies**

Anti-LC3/Atg8, anti-Ref2p, anti-RAB5, anti-RAB7, and anti-NPC antibodies were purchased from Abcam (Cambridge, MA, USA). Anti-Atg5 and anti-Atg12 antibodies were acquired from Cell Signaling Technology (Beverly, MA, USA). The anti-GAPDH antibody was from Santa Cruz Biotechnology (CA, USA). Rabbit anti-HA and anti-ubiquitin antibodies were from Beyotime Biotechnology (Shanghai, China). Mouse anti-HA and rabbit anti-c-myc antibodies were purchased from Sangon Biotech (Shanghai, China). Anti-Lamin, anti-Prospero, and anti-Delta antibodies were acquired form DSHB (IA, USA). Monoclonal Anti-HA−Agarose antibody produced in mouse was purchased from Sigma (CA, USA).

**Immunofluorescent staining**

Adult *Drosophila* intestines were dissected in phosphate-buffered saline (PBS) and fixed in 4% paraformaldehyde with 0.2% Triton X-100 (PBST) for 30 min. After blocking with 5% goat serum in 0.2% Triton X-100, samples were stained with primary antibody (1:200) at 4°C overnight, followed by incubation with secondary Alexa Fluor goat anti-rabbit or goat anti-mouse antibody (Abcam,1:1000) at 4°C overnight. Images were captured with a Leica SP5 confocal microscope. For visualization of cellular co-localization between ataxin3trpQ78 and other proteins, ImageJ (National Institutes of Health, USA) was used to obtain densitograms of ROI. For tri-colored co-localization analysis, a single cell area was selected using the freehand selection tool of image J, the color threshold adjusted, and the color channels of the selected area were split into red, green, and blue. Then two of the three color-maps were picked for colocalization-thresholds plugins analysis to obtain the two-channel co-localization map. After the color of the co-localization map was converted, the calculated co-localization map and the third channel picture were analyzed again to get the percentage of the triple-color area in the third channel.

**Immunoblotting and Immunoprecipitation**

*Drosophila* intestines were dissected at various times in PBS and lysed in 100 µl SDS lysis buffer (20 mM Tris/HCl at pH 7.6, 150 mM NaCl, 5 mM EDTA, 10% glycerol, 1% SDS and 1 × complete™, EDTA-free Protease Inhibitor Cocktail) for 30 min on ice. Intestine debris was eluted in 5 × SDS loading buffer and boiled for 10 min at 100°C. The supernatants from the centrifuge (12,000 g, 10 min) were separated by 12% SDS-PAGE, then transferred onto nitrocellulose membranes (Pall, NY, USA). Membranes were blocked with 5% BSA in TBS-T, incubated overnight with mouse anti-HA (1:1000, Sangon Biotech), mouse anti-GAPDH (1:1000, Santa Cruz), or rabbit anti-Atg12 antibody at 4°C, followed by incubation with HRP-conjugated secondary antibody (Beyotime) for 2 h at RT. Chemo-luminescence was detected with BeyoECL Plus reagent (Beyotime) and visualized using the ChemiDocTM XRS+ (BIO-RAD, Hercules, CA). For immunoprecipitations, tissues were grounded in lysis buffer (50 mM Tris-HCl pH 7.4, 150 mM NaCl, 1% Triton, 10% glycerol, 1 mM EDTA, and protease inhibitor cocktail). Cell lysates were immunoprecipitated with an Anti-HA−Agarose antibody at 4°C overnight. Then the beads were washed three times with the lysis buffer.

**RNA extraction and profiling**

3-d-old *Drosophila* intestines were dissected in PBS, and total RNA was extracted using RNAiso Plus (TaKaRa, Dalian, China). We assessed the quality of RNA samples using spectrophotometry. This involved examining A260/A280 ratio to ensure high-quality RNA suitable for downstream applications. The samples that met our quality criteria were selected for further gene expression profiling at the Beijing Genome Institute (BGI, Shenzhen, China).

**Quantitative real-time PCR**

The quantitative measurement of chaperones and Atgs levels in 5-d-old adult flies was carried out by real-time PCR using a Roche LC480 LightCycler instrument (Roche, Mannheim, Germany) with the UtraSYBR PCR mixture (CWbio, Beijing, China) in a one-step reaction according to the manufacturer’s instructions. Primers were designed using the NCBI online primer-design tool. The primer sequences used are listed in Supplementary Table 2. The melting curves and gel electrophoresis of the end products were obtained to confirm the specificity of PCR. The mRNA levels of corresponding genes were normalized to the relative amounts of the housekeeping gene, 18s. Results are expressed as fold changes compared to those of controls. Significance was determined by a Student’s *t*-test for each comparison of two groups.

**Gut morphology assays**

Individual guts (N = 8 to 15) were visualized and captured using a Leica DM6000B imager. Full guts were scanned at ×10 magnification and then recomposed with Adobe Photoshop CS6 (San Jose, CA). Using ImageJ, gut lengths were measured by tracing from the cardia to the anus. The mean widths of individual guts were based on the average of three measurements taken along the guts.

**Feeding assays**

The feeding assay was modified from Kanyan Xu et al (Xu et al., 2008). At 8 am, groups of 5-d-old male flies of (20 flies per group) were starved for 1 h and fed with blue food for 2 h. After feeding, the flies were homogenized in 400μl of PBS. Absorbance of the blue dye was measured at 625 nm. For assessment of defecation, the number of blue feces on the wall of tubes per 20 flies within 2 h were measured and recorded.

**Transmission electron microscopy (TEM)**

Intestines were fixed in 2.5% glutaraldehyde and 1% osmium tetroxyde and embedded in Epon resin according to standard procedures optimized for *Drosophila* tissue(Takashima et al., 2011). Ultra-thin sections were stained with uranyl acetate and lead citrate. HT7800 TEM was used for observation. MVBs, lysosomes, and autophagosomes per 100 μm^2^ were counted in random sections. Endosome volume, and ER content were measured using ImageJ Software. At least eight cells were quantified for each sample. Immuno-gold labeling was conducted as in Speese et al. and mouse anti-HA antibody was used as a primary antibody (Speese et al., 2012).

**Quantification of cell size and nuclear size**

The cell or nuclear size of ECs was quantified as previously described (Chang et al., 2013).

**External eye microscopy.**

For eye pictures in Supplementary Fig.S7, we used a Leica M205 FA motorized zoom microscope system with DFC295 digital camera and Leica Application Suite Montage module software (Leica Microsystems).

**Statistical analyses**

Statistical calculations were performed with Prism 6.0 (GraphPad Software, La Jolla, CA, USA) statistical software. All quantified results were from three independent experiments. To determine the percentage of cells containing each phenotype, randomly chosen ECs from more than ten views of at least 4 *Drosophila* intestines were counted in each experiment. For experiments analyzing fluorescence intensities, the mean values from individual cells from independent experiments were analyzed. Quantification of relative aggresome content was performed by calculating the mean aggresome area to the total cell/nucleus/cytoplasm area. Data are expressed as mean ± SD. unless otherwise indicated. Two treatment groups were compared by a Student’s *t*-test unless otherwise indicated and One-way ANOVA was used for multiple comparisons. Results were considered statistically significant when *P* < 0.05. All values represent mean ± SD. **p* < 0.05, ***p*< 0.01, ****p* < 0.001, **** *p*< 0.0001.

**Supplementary information**

**Supplementary Fig. S1**

**
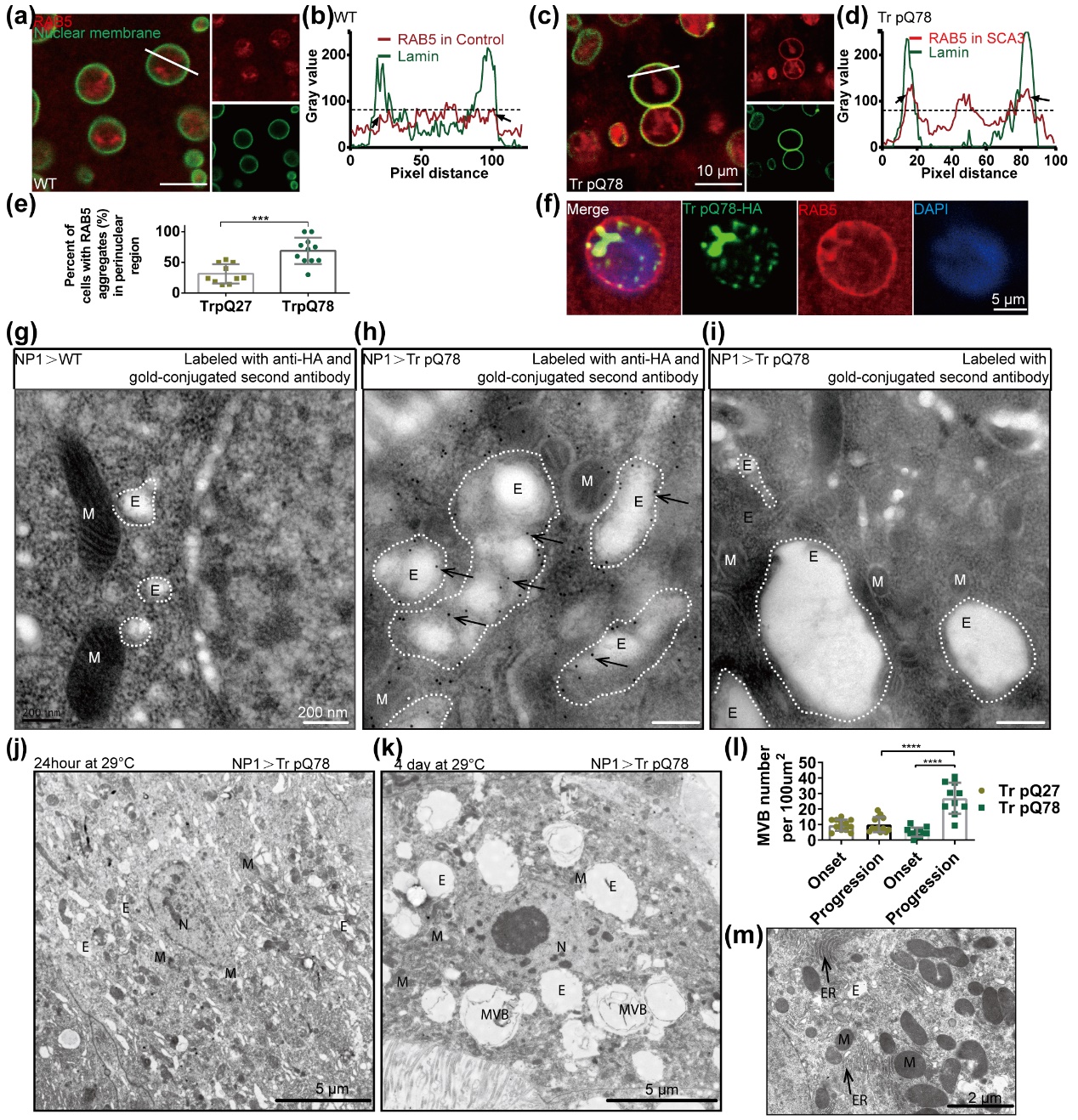
**

**Supplementary Fig. S1** Aberrant endosomes accompanied the disease progression in trpQ78-expressing ECs. **a–****d** Distribution of RAB5 in wild-type or trpQ78 (truncated ataxin3pQ78)-expressing ECs. RAB5 and nuclear membrane were stained with anti-RAB5 (red) and anti-Lamin (green), followed by densitogram analysis of the selected region of interest (ROI, white line). In trpQ78 group, the RAB5 signal colocalized with the nuclear lamin and reached deep into the center of the nucleus, shaped like a tunnel. **e** TrpQ78-expressing increased RAB5 aggregates in the perinuclear region (****p* < 0.001 from a student’s *t* test). **f** Tunnel-like RAB5 signal colocalized with intranuclear trpQ78 aggregates. **g** Immunoelectron micrograph of a wild-type EC labeled with anti-HA and gold-conjugated second antibody. M = mitochondria; E = endosome. **h** Immunoelectron micrograph of a trpQ78-HA expression EC labeled with anti-HA and gold-conjugated second antibody, trpQ78 signals were indicated by black arrowheads. **i** Immunoelectron micrograph of a trpQ78-HA expression EC labeled with the gold-conjugated second antibody only, as the negative control. **j** At the onset of the disease (< 24 h after eclosion), extensive small tubular or spherical endosomes were generated and widely distributed in the cytoplasm. **k** As global pathology became visible ( 3-5 d old adults undergo rapid disease progression, so this stage is defined as the disease progression ), endosomes were enlarged and accumulated in the juxtanuclear region, with a large number of MVBs (multiple vesicular bodies). **l** MVB number increased in trpQ78-expressing ECs with disease progression (*****p* < 0.001 from a one-way ANOVA). **m** TEM of ECs of 3-d-old wild-type genetic background control *Drosophila*. All values represent mean ± SD.

**Supplementary Fig. S2**


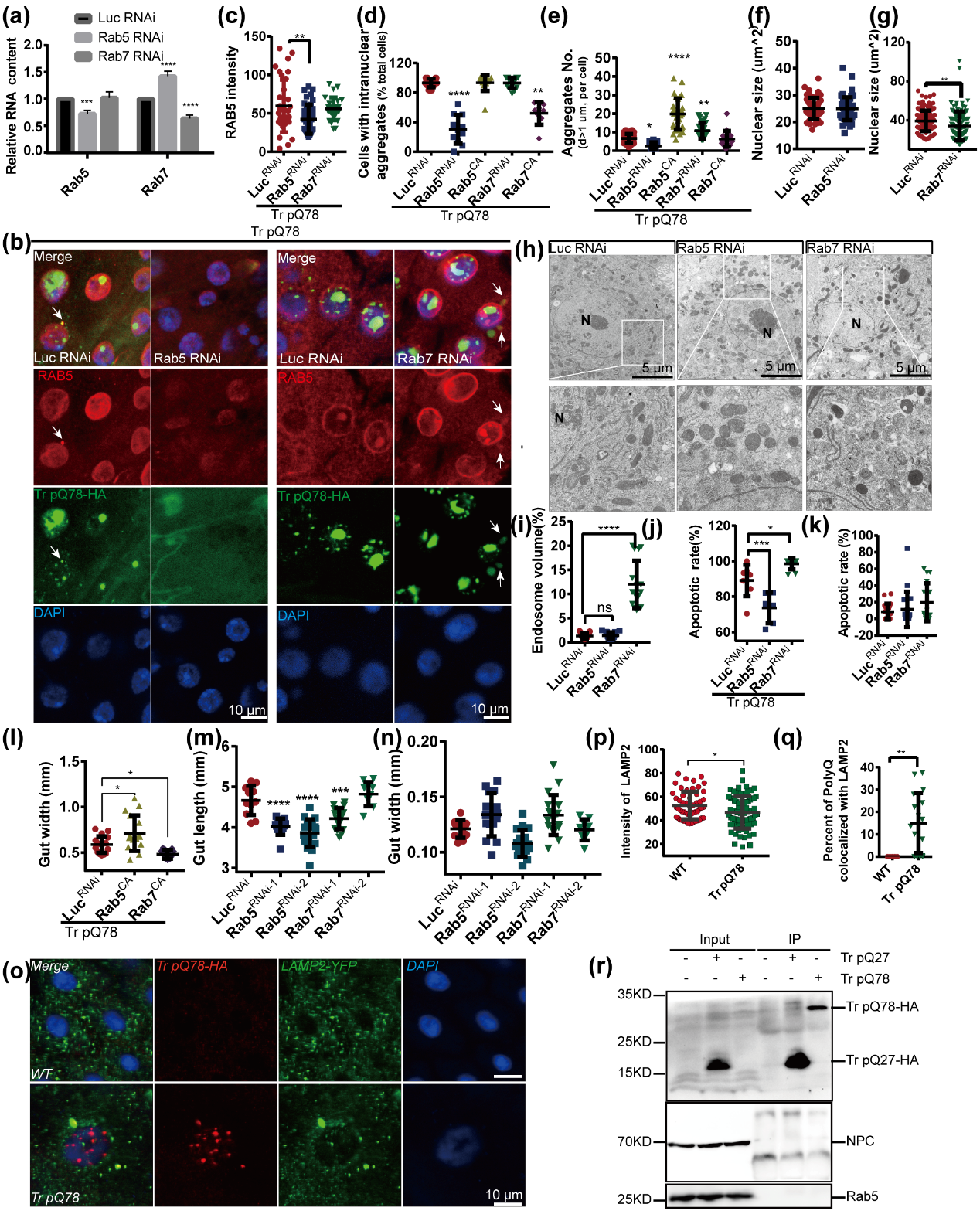


**Supplementary Fig. S2** Modifying effects of *Rabs* gene manipulation in the trpQ78 *Drosophila* midgut model. **a** Quantification of relative mRNA content of *Rab*5 and *Rab*7 in the fly intestines with indicated genotypes. Note that *Rab5* RNAi also up-regulated the transcription of *Rab7*. (****p <* 0.0001, *****p <* 0.0001 by student’s *t*-test). **b** TrpQ78 (truncated ataxin3pQ78) and RAB5 in ECs (enterocytes) with the indicated genotypes were visualized by anti-HA (green) and anti-RAB5 (red) immunofluorescence staining. The white arrows indicated the co-localized aggregates of polyQ and RAB5. Nuclei were visualized by DAPI staining. 4~5 intestines were randomly chosen from each group, and 3 fields of view were randomly selected for each intestine. **c** RAB5 signal could be inhibited by *Rab5* RNAi but showed no change in *Rab7* RNAi group. (***p <* 0.01 by One-way ANOVA). **d** Percentage of cells with intranuclear aggregates increased in Rab5^CA^ and *Rab7* RNAi groups in the trpQ78 context. The intranuclear area referred to the region surrounded by the lamin signal in Figure 3A. (***p <* 0.01, **** *p <* 0.0001 by One-way ANOVA). **e** *Rab5* RNAi and Rab7^CA^ decreased while Rab5^CA^ increased the number of cellular aggregates in the trpQ78 context. ECs containing at least one aggregate in Figure 3A were selected for quantification. (**p <* 0.05, ***p <* 0.01, *****p <* 0.0001 by One-way ANOVA). **f** No significant change in nuclear size of ECs in *Rab5* RNAi group compared with *Luc* (*Luciferase)* RNAi group (by Mann-Whitney test). **g** *Rab7* RNAi decreased the nuclear size of control ECs. (***p <* 0.01 by Mann-Whitney test). **h, i** Representative TEM of wild-type ECs with *Luc* or *Rabs* RNAi. Bottom-row showed a partial enlargement of photos from the top-row. *Rab7* but not *Rab5* RNAi significantly increased endosome volume (n≥5, *****p <* 0.0001 by One-way ANOVA). **J** Elevated TUNEL apoptotic rate in *Rab7* RNAi flies in the trpQ78 context. (**p <* 0.05, ****p <* 0.001 by One-way ANOVA). **k** TUNEL analysis showed no significant difference in wild-type *Drosophila* without or with *Rabs* RNAi. (By One-way ANOVA). **l** RAB5^CA^ increased while RAB7^CA^ decreased gut width of trpQ78-expressing guts (**p <* 0.05 by One-way ANOVA). **m** *Rab5* and *Rab7* RNAi decreased gut length in wild-type *Drosophila* compared with *Luc* RNAi group (****p <* 0.001, *****p <* 0.0001 by One-way ANOVA test). **n** No alteration of gut width in wild-type *Drosophila* with *Luc* or *Rabs* RNAi (by One-way ANOVA test). **o, p** Reduced LAMP2 signal in trpQ78-expressing ECs. LAMP2-YFP and trpQ78 were stained with GFP (green) and anti-HA (red) antibodies, respectively. Nuclei were visualized by DAPI staining (**p* < 0.05 by Mann-Whitney test). **q** Only a small fraction of trpQ78 colocalized with LAMP2-YFP. **r** No protein-protein interactions between trpQ78 and RAB5 or nuclear pore complex (NPC). Anti-NPC, anti-RAB5, and anti-HA antibodies were probed in this blot.

**Supplementary Fig. S3**

**
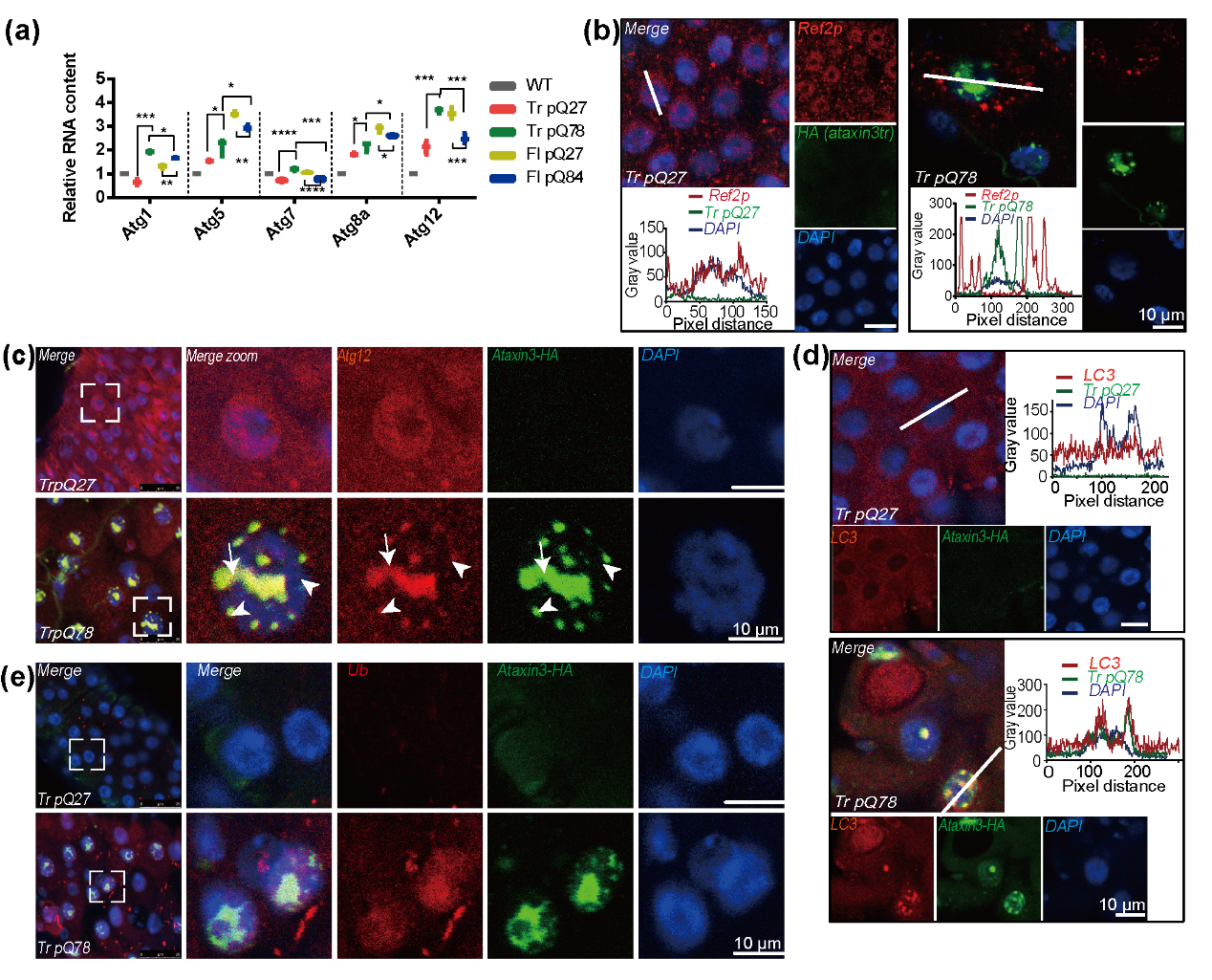
**

**Supplementary Fig. S3** TrpQ78 influenced the distribution pattern of Autophagy related proteins and ubiquitin. **a** mRNA levels of *Atgs* in the polyQ-expressing group and wildtype genetic background control. (*p *<* 0.05, ***p *<* 0.001, ****p *<* 0.0001 by student’s *t*-test). **b** Ref2p extensively co-localized with DAPI in the trpQ27 (truncated ataxin3pQ27)-expressing group but not in the trpQ78 (truncated ataxin3pQ78) group. Ref2p and ataxin3tr-HA were stained with anti-Ref2p (red) and anti-HA (green), respectively, followed by densitogram analysis of the selected region of interest (ROI, white line). **c** Atg12 colocalized with trpQ78 aggregates in trpQ78-expressing ECs. Atg12 and Ataxin3tr-HA were visualized by anti-Atg12 (red) and anti-HA (green) immunofluorescence staining, respectively. **d** Co-localization of Atg8/LC3 and trpQ78 aggregates. Atg8/LC3 and Ataxin3tr-HA were visualized by anti-LC3 (red) and anti-HA (green) immunofluorescence staining, respectively, and subjected to densitogram analysis of selected ROI. **e** Colocalization of ubiquitin (red) with ataxin3trpQ78-HA (green) in trpQ78-expressing ECs. Nuclei were visualized by DAPI staining.

**Supplementary Fig. S4**


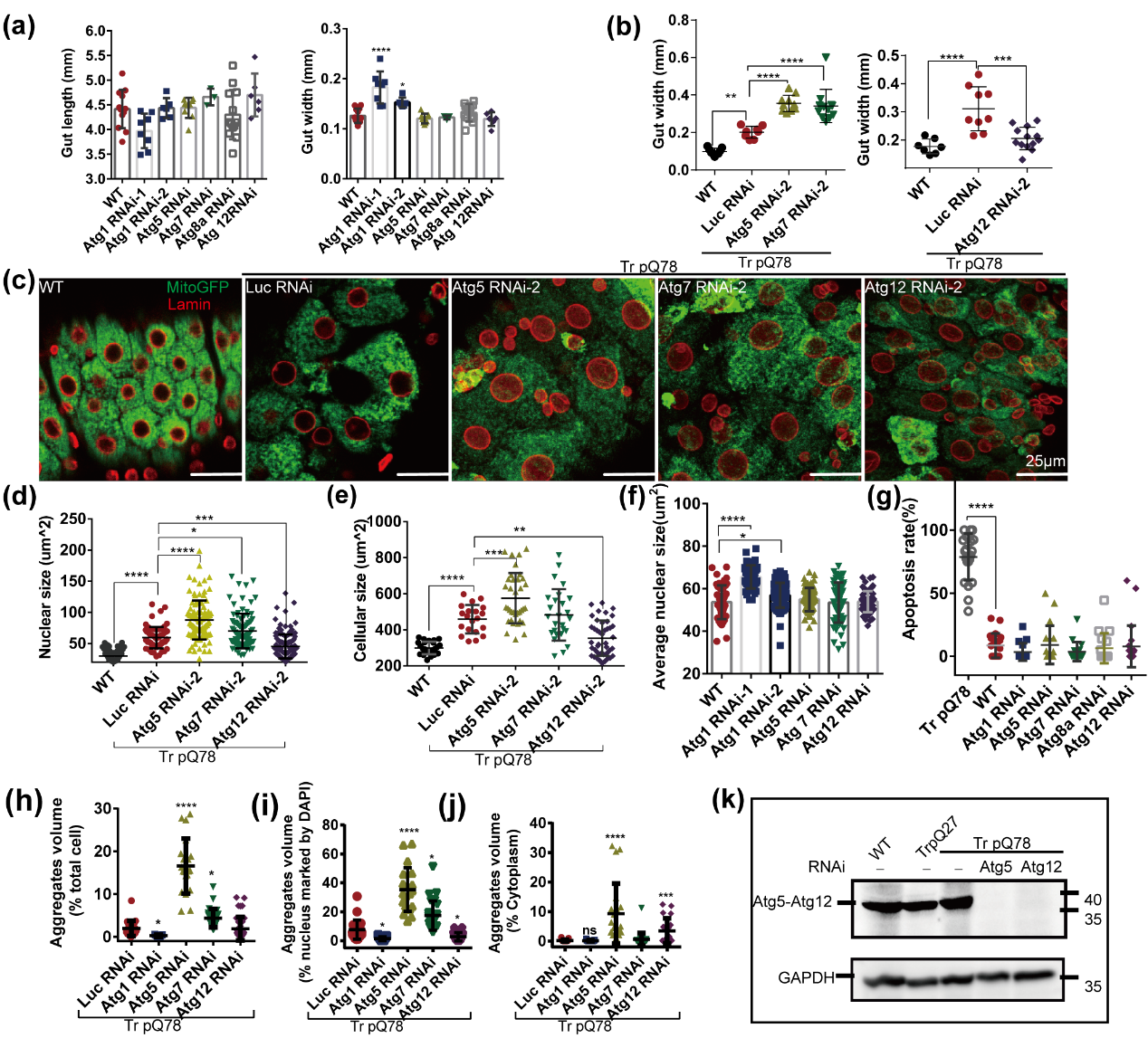


**Supplementary Fig. S4** Down-regulation of *Atgs* differentially modulated the SCA3 disease severity. **aa** *Atgs* gene manipulation alone had no significant influence on the gut morphology of background control *Drosophila* except minor impact by *Atg1* RNAi. Two independent *Atg1* RNAi lines were used. Independent Atg5-, Atg7-, Atg12- lines did not alter the global gut morphology (data not shown). **b** *Atg12* down-regulation restored the width of the guts and reduced gut-swelling, while *Atg5* and *Atg7* RNAi aggravated intestinal swelling in the trpQ78 (truncated ataxin3pQ78) context. The second *Atg5*, *Atg7*, *Atg12* RNAi lines were used. n≥8. **c** Confocal microscopy of cellular and nuclear outlines marked by MitoGFP and Lamin respectively in the SCA3 ECs with *Luc (Luciferase)* or *Atgs* RNAi. Two independent *Atg5* RNAi, *Atg7* RNAi and *Atg12* RNAi lines were used. Nuclei were visualized by DAPI staining. **d**, **e** Quantitative analysis of average cell size and nuclear size in SCA3 *Drosophila* without or with *Atgs* RNAi. **f** Quantitative analysis of average nuclear size in wild-type *Drosophila* without or with *Atgs* RNAi. **g** TUNEL analysis showed no significant difference among wild-type control genetic background *Drosophila* without or with *Atgs* RNAi. **h–j** Quantification of aggregates volume in the whole cell (**h**), the nuclear regions (**i**), and the cytoplasm (**j**). *Atg5* RNAi increased while *Atg1* or *Atg12* RNAi decreased the intranuclear content of trpQ78. **k** Western blotting confirmed the RNAi of *Atg5* or *Atg12*. Atg12-Atg5 complex did not increase in trpQ78-expressing intestines but decreased significantly with Atg5 or Atg12 RNAi. The blot was probed with an anti-Atg12 antibody. GAPDH was probed as a control. All values represent mean ± SD. **p* < 0.05, ***p*< 0.01, ****p* < 0.001, *****p* < 0.0001 by one-way ANOVA test (**a, b, d–j**).

**Supplementary Fig. S5**


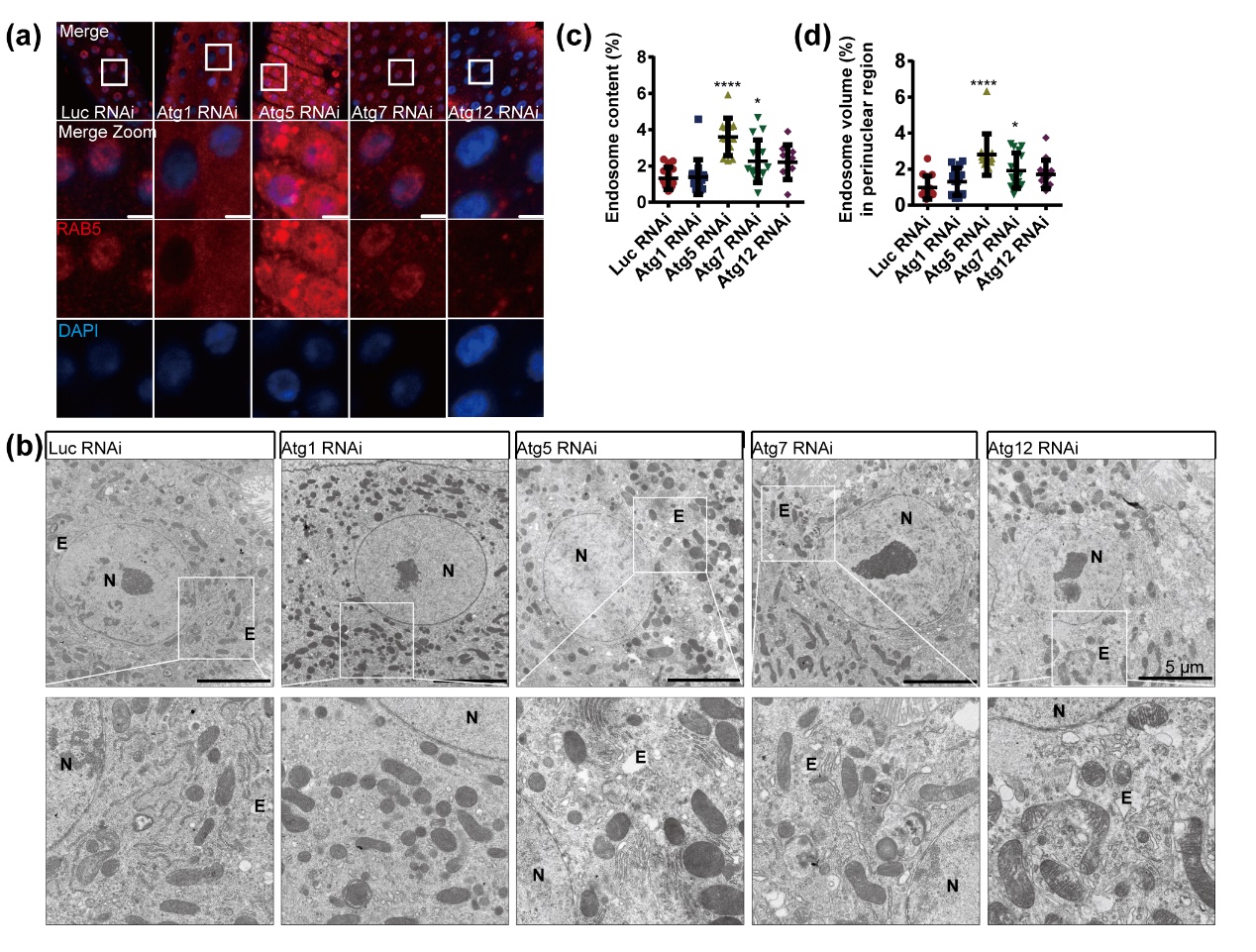


**Supplementary Fig. S5** ATGs affection on the volume and distribution of endosomes in the genetic background control. **a** RAB5 (red) influenced by different *Atgs* genetic manipulations in the genetic background control. *Atg5* and *Atg7* RNAi induced stronger RAB5 signal in ECs, with much more intensifying effect for *Atg5* RNAi. Nuclei were visualized by DAPI staining. For immunostaining test, 4~5 intestines were randomly chosen from each group, and 3 fields of view were randomly selected for each intestine. **b** Representative TEM of wild-type ECs without or with *Atgs* RNAi. Bottom-row showed a partial enlargement of photos from the top-row. **c**, **d** Endosome volume in the whole cell or perinuclear region (a region within a 2-μm radius of the outer nuclear membrane) of the genetic background control ECs without or with *Atgs* RNAi. n ≥5. By one-way ANOVA test. All values represent mean ± SD. *p < 0.05, **p < 0.01, ***p < 0.001, ****p < 0.0001.

**
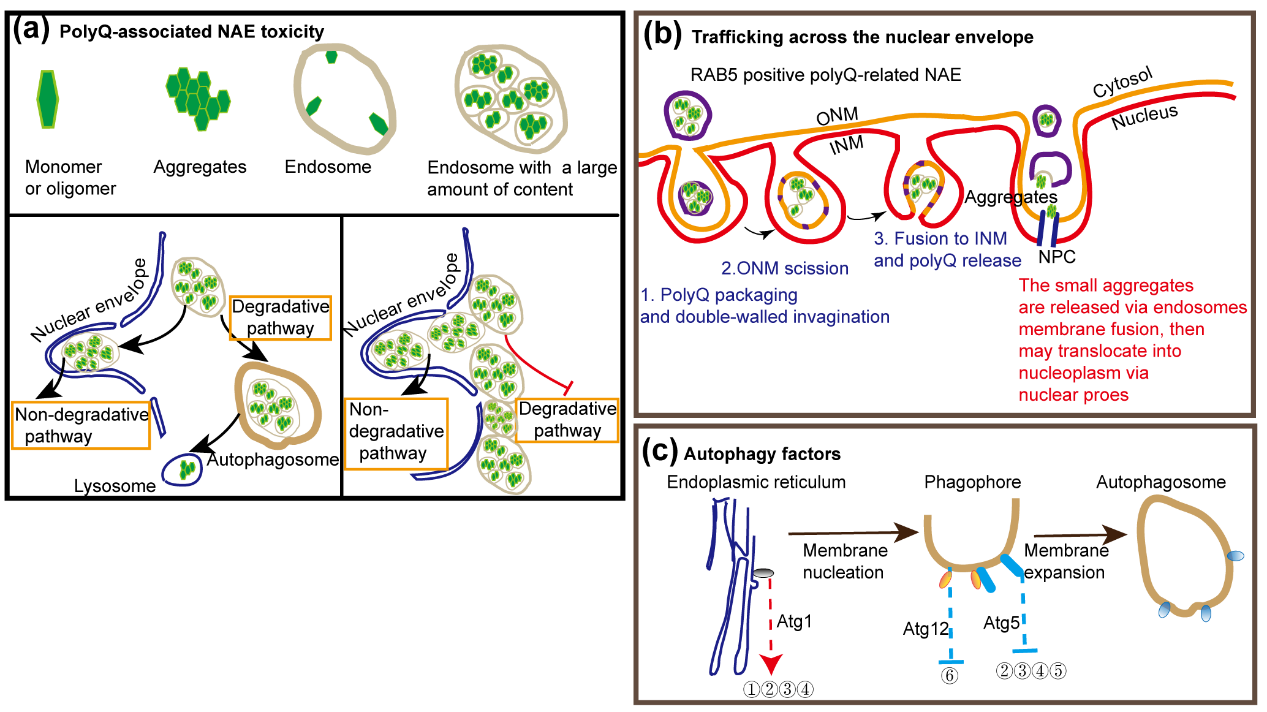
Supplementary Fig. S6**

**Supplementary Fig. S6** Schematic diagram of multiple routes centered on endosomes in regulating trpQ78 (truncated ataxin3pQ78) nucleocytoplasmic trafficking and toxicity. **a** Accumulated NAE and endomembrane disorganization contributed to trpQ78-mediated toxicity. The generation of polyQ monomers, oligomers, and aggregates, trpQ78-related NAEs in the pathogenesis of polyQ (top). In the early stages of the disease, both non-degradative pathway and endosome-autophagosome-lysosome degradative pathway are adopted for the cytoplasmic quality control of trpQ78 (left bottom). As the polyQ proteins accumulate, non-degradative pathway and impaired degradative pathway are insufficient to relief the polyQ-related NAE burden (right bottom). A large number of polyQ-related NAEs are accumulated around the nucleus, resulting in distorted endomembrane allocation and high toxicity. **b** A model for two potential nuclear ingress pathways (blue and red annotations) of trafficking trpQ78 aggregates to the nucleoplasm. The NPCs mediated pathway may only allow the passage of some small aggregates. As the number of NPCs decreases with the disease progression, this pathway may be defective and insufficient. **c** The interaction between autophagy factors and the endocytic pathway. Steps (1–5) in Fig.7 can be inhibited by Atg1 downregulation while steps (2–5) can be promoted by Atg5 downregulation. Step (6) in Fig.7 tends to occur upon Atg12 downregulation.

**Table 1**

| **RNAi** | **Cytoplasmic aggregates** | **Peri nuclear aggregates** | **Intranuclear aggregates** | **PolyQ related NAE number** | **PolyQ related NAE size** | **Severity of disease** | **TUNEL** |
| --- | --- | --- | --- | --- | --- | --- | --- |
| **Atg1** | **-** | **↓** | **↓** | **↓** | **↓** | **↓** | **↓** |
| **Atg5** | **↑** | **↑** | **↑** | **↑** | **↑** | **↑** | **↑** |
| **Atg7** | **-** | **-** | **↓** | **↑** | **-** | **↑** | **↑** |
| **Atg12** | **↑** | **↓** | **↓** | **↓** | **↓** | **↓** | **↓** |
| **Rab7** | **-** |  |  | **↑** |  | **↑** | **↑** |
| **Rab5** |  | **↓** | **↓** | **↓** | **↓** | **↓** | **↓** |

**Supplementary Table 1 The effect of gene knockdown on endosomal development, distribution of polyQ and SCA3 disease severity.** – (no change), ↑ (increase), ↓ (decrease), / (NA).

**Table 2**

| Gene name | Primer sequence | Size in bp |
| --- | --- | --- |
| Atg1 | F: GCCAGCTCCATCGAAAATAACC  R: GCGGCGCAGCAGGCACAG | 325 |
| Atg5 | F: GCCCCTGCGACTTCACTATCC  R: CCATTAAATCGGCCAAACTCTTCT | 434 |
| Atg7 | F: AGTATCCAACATTGCGGCCA  R: CTCTGTGCATAGTAGGCGGG | 96 |
| Atg8a | F: AGCCACAGCAGTTAGCGAG  R: TTGTGTAGAGTGACCGTGCG | 120 |
| Atg12 | F: AAAACTGAGGATCAAAGGAG  R: CAATAAGATGAACCAAGACA | 100 |
| Rab5 | F:ACCAGGAGAGCACGATAGGT  R: GCCTGCGCTCCTCGATAATA | 142 |
| Rab7 | F: ATCAACGTGGAGATGGCGTT  R: TGCGAGCCCAAGGTAATCTG | 107 |
| 18s | F:TCTAGCAATAATGAGATTGAGCAATAA  R:AATACACGTTGATACTTTCATTGTAGC | 93 |
| dCyck-gDNA | F: GAGCATCCTTACACCTTTCTCCT  R: TAATCTCCGGCTCCCACTG | 145 |
| COX1 | F: GTGCTCCTGATATAGCATTCCCA  R: CACCATGAGCAATTCCAGCG | 156 |
| COX3 | F:ACTGTAACTTGAGCCCACCAT  R: TTGAGTCTGCAATAGTAAATGGAGC | 148 |
| CYTB | F: AACACCTGCCCATATTCAACCA  R: TTCAACTGGTCGAGCTCCAAT | 244 |

**Supplementary** **Table** **2 RT-PCR conditions and primer sequences**

**References**

Chang, T.K., Shravage, B.V., Hayes, S.D., Powers, C.M., Simin, R.T., Wade Harper, J., and Baehrecke, E.H. (2013). Uba1 functions in Atg7- and Atg3-independent autophagy. Nat Cell Biol *15*, 1067-1078.

Speese, S.D., Ashley, J., Jokhi, V., Nunnari, J., Barria, R., Li, Y., Ataman, B., Koon, A., Chang, Y.T., Li, Q.*, et al.* (2012). Nuclear envelope budding enables large ribonucleoprotein particle export during synaptic Wnt signaling. Cell *149*, 832-846.

Takashima, S., Younossi-Hartenstein, A., Ortiz, P.A., and Hartenstein, V. (2011). A novel tissue in an established model system: the Drosophila pupal midgut. Dev Genes Evol *221*, 69-81.

Xu, K., Zheng, X., and Sehgal, A. (2008). Regulation of feeding and metabolism by neuronal and peripheral clocks in Drosophila. Cell Metab *8*, 289-300.
